# Global analysis reveals persistent shortfalls and regional differences in availability of foods needed for health

**DOI:** 10.1016/j.gfs.2024.100825

**Published:** 2025-03

**Authors:** Leah Costlow, Anna Herforth, Timothy B. Sulser, Nicola Cenacchi, William A. Masters

**Affiliations:** a Friedman School of Nutrition Science and Policy, Tufts University, USA; b Food Prices for Nutrition Project, Boston, USA; c Foresight and Policy Modeling, International Food Policy Research Institute (IFPRI), Washington D.C, USA; d Department of Economics, Tufts University, USA

**Keywords:** Healthy diets, Food-based dietary guidelines, Food systems, Food systems transformation, Climate change

## Abstract

Sufficient food is available in the world for all people to consume sufficient calories, but not healthy diets. This study traces historical and projected changes in global food systems toward alignment with the new Healthy Diet Basket (HDB) used by UN agencies and the World Bank to monitor the cost and affordability of healthy diets worldwide. Using the HDB as a standard to measure adequacy of national, regional and global supply-demand balances, we find substantial but inconsistent progress toward closer alignment with dietary guidelines, with large global shortfalls in fruits, vegetables, and legumes, nuts, and seeds, and large disparities among regions in use of animal source foods. Projections show that additional investments aimed at reducing chronic hunger would modestly accelerate improvements in adequacy where shortfalls are greatest, revealing the need for complementary investments to increase access to under-consumed food groups especially in low-income countries.

## Highlights

• Sufficient food is available in terms of calories, but not healthy diets.
• We compare quantities to Healthy Diet Basket targets used to monitor food access.
• Average shortfalls have declined from 44% in the 1960s to 28% in the 2020s.
• Given climate change, projections show modest further gains to 2050.
• Meeting health needs would require faster innovation to close food supply gaps.

## Graphical abstract

**Figure:**
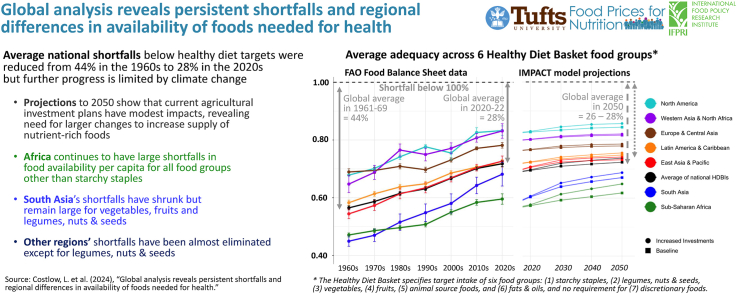

## 1 Background and motivation

International efforts to reduce the burdens of malnutrition and diet-related disease depend on increased supply of the food groups needed for healthy diets. New initiatives are moving beyond historical investments designed to meet dietary energy needs (Pingali, 2012, 2015), towards elimination of micronutrient deficiencies and inadequate diets among the most vulnerable (FAO, 2024a, FAO, 2024b, FAO, 2024c; Vos et al., 2020) and prevention of diabetes, hypertension, and other diseases (Abarca-Gómez et al., 2017; Popkin et al., 2012). Poor diets and malnutrition are the world's leading modifiable risk factors for death and disability, with suboptimal diets resulting in 11 million premature deaths globally in 2017 (Afshin et al., 2019).

Governments often use food-based dietary guidelines (FBDGs), produced by national health authorities to summarize the evolving scientific consensus, to define healthy diets and help inform consumers on which food groups to eat and in what proportions to maintain energy balance, meet nutrient requirements, and protect against noncommunicable diseases (FAO, 2023). There is substantial agreement among FBDGs, recommending consumption of fruits, vegetables, starchy staples, animal-source foods, oils and fats, and legumes, nuts and seeds in appropriate proportions (Herforth et al., 2019), and there is clear evidence that dietary patterns around the world fall short of these recommendations (Global Diet Quality Project, 2022; Miller et al., 2022).

Previous studies have addressed how global food supplies reach or fall short of requirements for essential nutrients (Fletcher et al., 2024; Lividini and Masters, 2022; Wang et al., 2023), specified food groups (fruits and vegetables, sugar, red and processed meat, fish and seafood) (Clifford Astbury et al., 2021; Mason-D’Croz et al., 2019a), or other reference diets including the EAT-Lancet dietary patterns or the Harvard Healthy Eating Plate (Tuninetti et al., 2022; Krishna Bahadur et al., 2018). Each of these studies finds shortfalls in fruits and vegetables; shortfalls of other food groups where examined (e.g. legumes and nuts according to Tuninetti et al., 2022, and protein and milk, according to Krishna Bahadur et al., 2018); shortfalls for numerous vitamins and minerals including calcium, vitamin A, vitamin E, and vitamin B12; and excesses of sugar, starchy staples, meat, and dietary energy. Large-scale food fortification may play an important role in reducing micronutrient deficiencies and related diseases, as micronutrient intakes are inadequate for more than 60% of the world's population (Passarelli et al., 2024). Micronutrient interventions, however, compensate for the underlying problem which is lack of diverse, healthy diets. There is not yet a comprehensive picture of how quantities produced and consumed around the world align with total diets composed of the food groups specified in national dietary guidelines.

Since 2022 the new global Healthy Diet Basket (HDB) has been used by UN agencies and the World Bank for global monitoring of the cost and affordability of a healthy diet (FAO, 2024a; The World Bank, 2024). The HDB specifies benchmark quantities across six food groups (starchy staples; fruits; vegetables; animal-source foods; legumes, nuts, and seeds; and oils and fats), expressed in terms of dietary energy per day, and representing common requirements among national FBDGs (Herforth et al., 2022, 2024). The UN agencies use it for monitoring the Cost of a Healthy Diet (CoHD) indicator because it is the first reference diet that has been explicitly derived from FBDGs that countries around the world have published, reflecting broad international consensus in healthy diet patterns. As in all FBDGs, the food groups are constant as generally universal components of a healthy diet, but there is wide variability in the locally-available food items that can satisfy them (for example, groundnut sauce, dal, and hummus all fit in the legumes, nuts, and seeds group). Therefore within each food group, any combination of items may be consumed according to availability and preferences, allowing for local and regional variation in diets. The HDB composition is broadly similar to all national FBDGs and other reference diets such as the Harvard Healthy Eating Plate (analyzed in Krishna Bahadur et al., 2018), but we use the HDB because it is used by the UN agencies for global monitoring of access to healthy diets since 2022. Diet baskets meeting HDB quantity benchmarks have adequate macronutrient balance and a mean adequacy ratio of 95% for micronutrients and protein for the reference person of a healthy adult woman (Herforth et al., 2024). National guidelines and other recommendations often specify additional targets, such as those for intakes of whole grains, salt, and sugar (Herforth et al., 2019); of these, food supply data are amenable to comparison against international guidelines on sugar consumption (WHO, 2015).

This paper provides the first global analysis of food availability relative to a single benchmark for healthy diets that reflects the international consensus across dietary guidelines. We compare historical and projected food supply data with the reference quantities for each HDB food group. This analysis shows the extent to which adequate quantities of food exist in the food supply for all people to meet dietary needs in each country and year, under optimistic conditions of equitable distribution and access within countries across diverse populations and subnational regions. For historical data on quantities, we look to FAO food balance sheets (FBS) since 1961. For future projections, we build on the historical evidence from FBS by incorporating economic modeling projections from the International Model for Policy Analysis of Agricultural Commodities and Trade (IMPACT) (IFPRI, 2023). IMPACT is a modeling framework developed by IFPRI to simulate changes in agricultural markets under a variety of scenarios for major food system drivers, such as climate change, technology, and socioeconomic changes. Our food supply projections derive from an IMPACT modeling exercise designed to estimate the cost of investments required to counteract the rise in chronic hunger that would otherwise occur under climate change, as measured by the prevalence of undernourishment (Sulser et al., 2021). This modeling framework operates on a partial equilibrium basis, which means it does not account for connections between agricultural markets and other economic sectors. We analyze food supply projections that compare business-as-usual trends in agricultural investments with broad-based changes in investment patterns to counteract chronic hunger in low- and middle-income countries. Food supply projections under the business-as-usual scenario reflect the likely outcome in the absence of a global effort to mitigate the effects of climate change on the most vulnerable populations around the world. Projections under the increased investment scenarios reflect the potential downstream consequences of investments targeting hunger, without investments covering a greater diversity of foods or repurposing policies toward increasing access to healthy diets.

## 2 Data and methods

### 2.1 Food balance sheets

We use food supply data from the FAO FBS, beginning with data from 153 countries in 1961 and ending with data from 188 countries in 2022 (FAO, 2024b, 2024c). Food supply is calculated as the daily per capita energy (using population data compiled by FAO) available in each country and each year, beginning with domestic production and accounting for imports net of exports, pre-retail loss, changes in stocks, and non-food uses such as feed, seed, and industrial applications.

We match each FAO commodity to its corresponding HDB food group and combine some FAO commodities into aggregate groups when the quantity available is small. For example, yams are added to the pre-existing group “other roots”, while demersal fish, pelagic fish, and other marine fish are combined into the new group “other marine fish” (See Supplementary Table S1 for a complete list of these groupings.). We obtain regional aggregates by adding national availability by commodity and HDB food group for each subregion in the United Nations geoscheme and combine subregions to reach 7 total world regions. RStudio (version 2024.04.1 + 748) was used to combine the old and new FBS databases and to perform all data analysis and visualizations. R code is available to replicate and update the results presented here with future annual updates to the FBS database. Country inclusion as well as starting and ending years for each country are described in Supplementary Table S2, while regional data are shown in Table S3.

### 2.2 Healthy Diet Basket and Healthy Diet Basket Index (HDBI)

The HDB specifies reference intakes for six food groups in recommended amounts derived from numerous national FBDGs, calibrated to meet the energy needs of a reference adult woman (Table 1). Free sugars are not included in the HDB, so we assess sugar supply using the WHO guideline on limiting free sugar intake to less than 10% of total dietary energy (WHO, 2015).

**Table 1:** Healthy Diet Basket (HDB) amounts per capita.

Food Group	Target intake (kcal/day)
Starchy staples	1160
Fruits	160
Vegetables	110
Animal-source foods	300
Legumes, nuts, and seeds	300
Oils and fats	300
Total	2330

To construct a Healthy Diet Basket Index (HDBI), each country or region's average shortfall below HDB targets summarizes the extent to which national, regional, or global food systems provide sufficient foods to meet recommended quantities in national dietary guidelines. Over the six recommended food groups *i*, we report the average degree to which available quantities $q_{i}$ fall below the HDB target $Q_{i}$, expressed as the absolute value of proportional shortfalls from 0 to −1 for food groups where $q_{i}$ is below $Q_{i}$. Our index of adequacy is 1 minus the average shortfall over all six food groups:

(1) $$HDBI=1-\frac{1}{6}\sum_{i=1}^{6}\left|\frac{q_{i}-Q_{i}}{Q_{i}}\right|\;\forall \;q_{i}<\;Q_{i}$$

This metric is analogous to the mean adequacy ratio used for essential nutrients, as shortfalls below targets for each food group are equally weighted and not offset by excesses of any food group. The highest attainable adequacy score of 1 represents a benchmark situation with zero shortfalls, while an adequacy score of 0 would be a hypothetical extreme where all energy was obtained from sugar. Possible scores indicating sufficient energy but limited food supply would include 0.167 for a scenario where all energy requirements are met from starchy staples alone, or 0.333 if all energy is from combinations of starchy staples and pulses such as chapati and dal, maize and peanut stew, or rice and beans, for example. A score of 0.833 could arise if four different food groups are used at or above target levels and the two remaining groups are consumed at 50 percent of their HDB target.

The shortfall approach is calculated under an assumption of perfect distribution. This assumption is never true, so in reality a higher amount than the per capita target would be needed for all people to access healthy diets, given that some people will overconsume. However, there is no clear method for determining the buffer amount of each food group that would need to be available in the food supply to account for imperfect distribution. Therefore we use the per capita daily recommended amounts as an absolute lowest bound on adequate supply, with the interpretation that if supplies are below adequate amounts in a perfect distribution scenario, then they are certainly below adequate amounts for all to access healthy diets under conditions of imperfect distribution. The HDBI shortfalls therefore are the most conservative description of shortfalls, and actual shortfalls would be greater by an undefined amount due to imperfect distribution.

### 2.3 Projected HDBI scores with the IMPACT model

Full IMPACT model specifications are discussed at length in related publications (Mason-D’Croz et al., 2019b; Robinson et al., 2015; Rosegrant et al., 2017; Sulser et al., 2021). In this study we include two scenarios which capture possible future investments in agricultural R&D. The “reference” (or business-as-usual) scenario projects forward historical investment trends made by national agricultural research systems and the research centers unified within the Consultive Group on International Agricultural Research (CGIAR), resulting in an estimated 2.5 to 3 percent annual growth in investments. The “increased investments” scenario models increased CGIAR investments that are designed to counteract the increase in chronic hunger under climate change. These include investments in (1) international research and development (R&D) for genetic improvement and accompanying management changes in specific commodities in some food groups in proportion to past R&D investments; (2) infrastructure and institutional change for reduced marketing margins; (3) expanded and more efficient irrigation; and (4) technologies leading to improved soil quality through better water-holding capacity (for individual scenarios see Supplementary Fig. S1). Agricultural R&D investments are calibrated to close yield gaps for cereals, roots, tubers, plantains and bananas, animal-sourced foods, pulses, and oilseeds, and do not include vegetables or fruits (other than plantains and bananas). Subsequent changes in the availability of vegetables and fruits are modeled from increased incomes and other compounding effects linked to increased productivity in the targeted crops. The investments are focused on target countries for the CGIAR and therefore exclude high-income regions.

Both scenarios are modeled under pessimistic conditions for both climate impacts and socioeconomic outcomes. The RCP8.5 climate change scenario assumes limited reductions in greenhouse gas emissions, and the SSP3 or “Regional Rivalry” shared socioeconomic pathway assumes relatively high population growth and relatively low income growth. Under these near-worst-case conditions, the IMPACT model estimates the magnitude of investments required to prevent new increases in chronic hunger in CGIAR focus countries. The model holds current trends constant in most wealthy countries and estimates the results of increased agricultural investments in most low- and middle-income countries. We calculate the percentage change in each food group's availability under each investment scenario and estimate HDBI scores by applying projected changes to the observed FBS availability of each food group in each region in the IMPACT reference year of 2010.

A total of 158 countries and country aggregates are included in the IMPACT model. Other than for a few aggregate regions (e.g. for small island regions), there is a close correspondence between IMPACT and FBS country inclusion. For Fig. 4, we use 2010 population data to calculate food group totals for IMPACT country aggregates when all countries within an IMPACT country group are included in FBS data. This analytical step is possible for the IMPACT country aggregates Baltic States, Other Balkans, Other Southeast Asia, and Rest of Arab Peninsula, but is not possible for country aggregates including numerous small island territories (see Supplementary Table 5).

## 3 Results

### 3.1 Past trends

Food systems provide varying levels of total dietary energy, as shown in Table 2. These data represent the sum of each population's food use, from estimated production plus imports and minus exports, non-food uses and losses prior to household consumption. Data are transformations of FAO Food Balance Sheet estimates for 1961 to 2022 from national government data (FAO, 2024b, 2024c), adjusted for region population and expressed in terms of daily energy available per capita in each year. Totals shown in the table exceed dietary intake due to kitchen and plate waste or other uses, and dietary intake itself varies with height, weight and physical activity.

**Table 2:** Daily energy available in kilocalories per capita globally and by region, 1960s and 2020s.

Region	1960s	2020s
East Asia & Pacific	1822	3096
Europe & Central Asia	2963	3224
Latin America & Caribbean	2278	2986
North America	2792	3691
South Asia	2004	2532
Sub-Saharan Africa	1982	2385
Western Asia & North Africa	2242	3066
World	2225	2881

Note: Data shown are the mean regional and worldwide totals from 1961 through 1969 (1960s) and from 2020 through 2022 (2020s), adding up total supply including the six HDB food groups plus sugars. Remaining commodities including spices, infant foods, beverages, and stimulants are excluded from these totals.

**Fig. 1:**
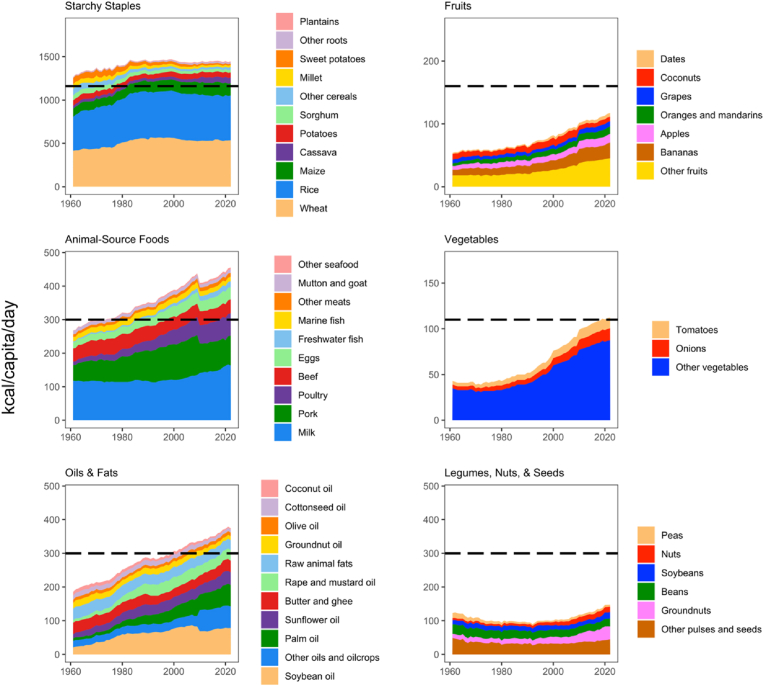
Global food supply relative to Healthy Diet Basket targets, 1961-2022 Note: Data shown are worldwide totals for food supply (kcal/capita/day), computed by the authors from national data in FAOSTAT. Plots are scaled to reference intake values. Black dashed lines indicate Healthy Diet Basket targets used to measure access to a healthy diet as recommended in national dietary guidelines.

The composition of aggregate global food supplies in terms of the six HDB food groups is shown in Fig. 1.

The per capita global availability of all six food groups has increased in different ways. For starchy staples, global supplies rose above the target for a balanced diet in the 1960s and 1970s but have not increased since the mid-1980s. The HDB amount of animal source foods was reached in the 1970s and supply has continued to rise, exceeding the target amount. The supply of oils and fats has continued to rise beyond the HDB target, which was reached in the early 2000s. Fruit and vegetable supplies saw little or no increase in the 1960s and 1970s but have grown sharply since then, especially for vegetables, while supplies of legumes, nuts, and seeds actually declined through the 1960s and 1970s before stabilizing in the 1980s and 1990s and rising slowly since the 2000s. Additionally, the composition of each food group has changed over time in important ways. For starchy staples, wheat and rice remain dominant, while animal source foods are increasingly provided by milk, poultry, and pork. The most dramatic shift is the increasing role of palm and soybean oil, as well as bananas among fruits, onions, tomatoes, and other vegetables. Among legumes, nuts and seeds, groundnuts have increased but the diverse groups of “beans” and “other pulses and seeds” have declined in per capita availability since 1961.

**Fig. 2:**
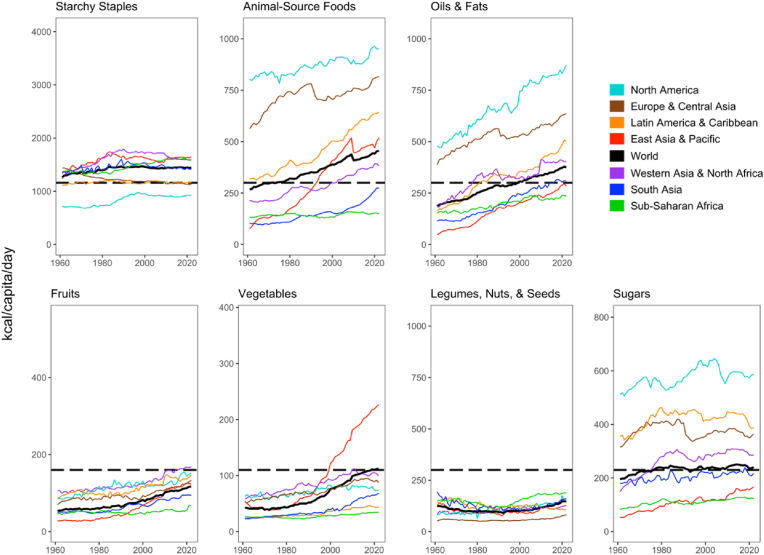
Regional food supplies relative to Healthy Diet Basket targets, 1961-2022 Note: Data shown are worldwide totals for daily per capita kilocalories available as food, computed by the authors from national data in FAOSTAT. Plots are scaled to reference intake values. Black dashed lines indicate Healthy Diet Basket targets used to measure access to a healthy diet as recommended in national dietary guidelines. Sugar availability is shown in reference to the WHO guideline that sugar intake be limited to less than 10% of total dietary energy (WHO, 2015).

Some trends in food availability by food group have been similar among major world regions, with important differences as shown in Fig. 2.

Food groups and regions differ in both levels and changes over time, often with sustained plateaus or periods of either decline or rapid increase. Notable declines in availability include starchy staples in East Asia and the Pacific since their peak in the early 1980s, and in Europe and Central Asia, where supplies declined through the 1960s and 1970s before stabilizing near the HDB target. Supplies of legumes, nuts and seeds are below the HDB target in all regions and declined further in several regions before rising in Sub-Saharan Africa since the 1990s. Availability of sugars rose in the 1960s and 1970s and then declined or plateaued in all regions except North America, where it continued to rise through the 1990s, and East Asia and the Pacific, where sugar continues to rise but remains well below the WHO's recommended limit.

Shortfalls in supply of healthy food groups below the HDB targets remain severe in several regions. For Sub-Saharan Africa, only starchy staples are available in adequate quantities, and only vegetable oils as well as legumes, nuts and seeds have increased significantly towards their HDB target. In South Asia, quantities of vegetables and fruits have increased, and oils and fats recently reached the HDB target in the late 2010s; while quantities, of sugars have stayed near the WHO limit since the 1980s, starchy staples have plateaued since the 1990s, and the legumes, nuts and seeds group declined from the world's highest levels in 1961 down to the global average and have barely risen since 2000. East Asia and the Pacific has had rapid growth in use of all food groups except starchy staples, and legumes, nuts and seeds, with availability rising above HDB targets since 1990 for animal source foods and since 2000 for vegetables, driven by extremely fast growth in vegetable availability in mainland China since the 1980s (see Supplementary Fig. S2). In North America, starchy staples have remained constant since the 1990s at a level slightly below the target intake, while animal source foods, oils and fats, and sugars have remained at levels two or even three times higher than the target intake, suggesting that increased consumption of these food groups has more than compensated for this small deficit in energy intake from starchy staples.

**Fig. 3:**
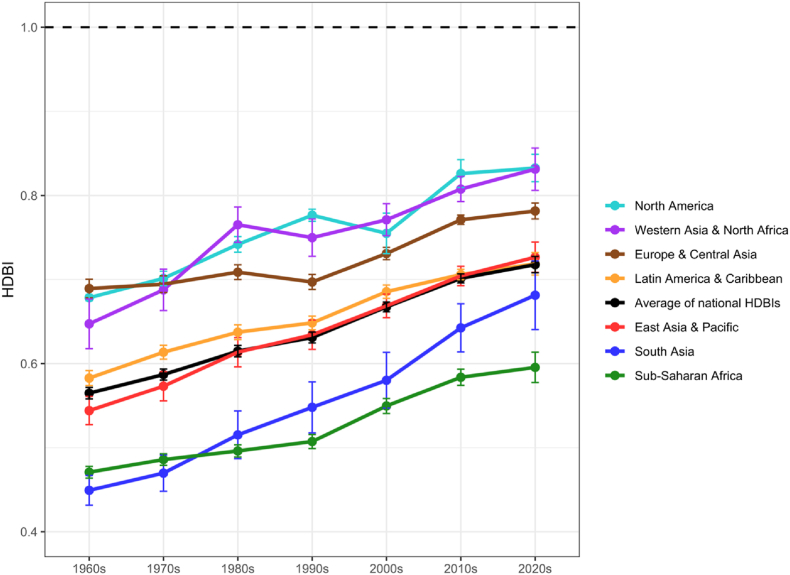
HDB index scores for adequacy of national food supplies by region and decade, 1960s-2020s Note: Points show means of all countries' average proportion of target availability for the six Healthy Diet Basket (HDB) food groups, by geographic region and decade. Error bars indicate 95% confidence intervals. The black dashed line indicates the reference level where all food groups are supplied at or above the HDB target. The decade labelled as “2020s” includes data from 2020 through 2022.

To describe results over all food groups, we compute for each country the average percentage deficit over all food groups whose supply is at or below its HDB target. This computation produces a Healthy Diet Basket Index (HDBI), which summarizes the level of adequacy with respect to a diet needed for long-term health (HDBI = 1 for complete adequacy across all six food groups). Fig. 3 reports the mean level of adequacy over all countries in each region for each decade. For example, national food supplies in South Asia had an average HDBI of 0.45 in the 1960s, indicating that only 45% of HDB benchmarks were met. In the 2010s, this number rose to 0.65, representing an improvement of 20 percentage points towards food supply alignment with dietary guidelines.

**Fig. 4:**
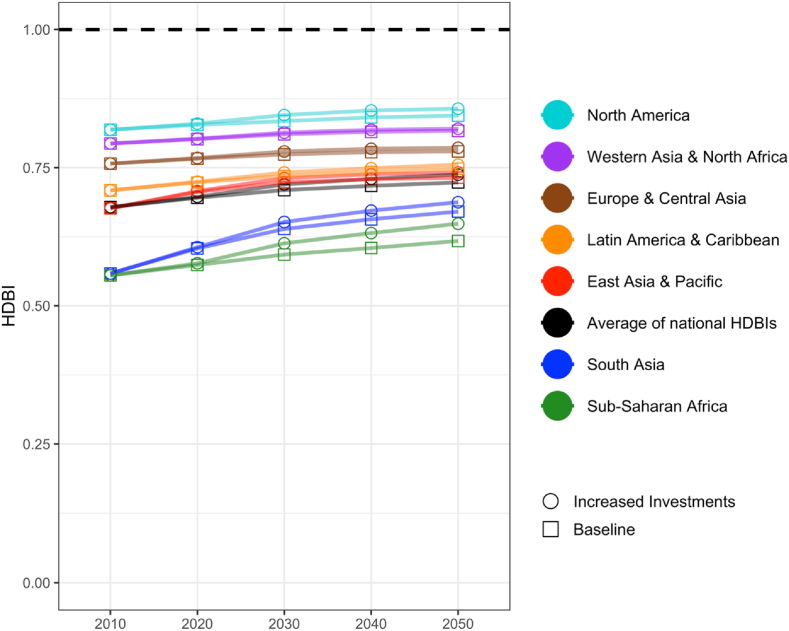
Projected HDB index scores for adequacy of national food supplies by region, 2010-2050 Note: Points show means of all countries' average proportion of target availability for the six Healthy Diet Basket (HDB) food groups, by geographic region and decade. Solid squares show baseline investment levels and solid circles show outcomes from an increased investments scenario. All outcomes are IMPACT model estimates given the SSP3 shared socioeconomic pathway and RCP8.5 climate-change scenario, using IMPACT to compute annual percentage changes in daily energy per capita of each commodity available for food use from the base year of observed quantities in 2010. Country inclusion described in the methods section and in Supplementary Table 5.

The data in Fig. 3 show how shortfalls below HDB targets are gradually closing over time, with faster progress in some regions than others. Average shortfalls across the food groups worsened during the 1980s in two regions (Europe and Central Asia, as well as Western Asia and North Africa) and worsened in the 2000s in North America, but otherwise have improved over time, especially in East Asia since the 1960s, South Asia since the 1970s, and Sub-Saharan Africa since the 1990s.

### 3.2 Modeled projections

Trends for adequacy of the food supply will be affected by strong socioeconomic pressures (e.g., population and income growth) and by climate change, but they will also respond to policy changes. Fig. 4 shows how future food adequacy could change between 2010 and 2050, using model simulations extracted from the IMPACT model (Sulser et al., 2021). Fig. 4 shows projections based on a business-as-usual scenario and one scenario of increased investments in a limited set of commodities and improved water use.

The IMPACT model's baseline for 2010 and 2020 differs somewhat from the average of countries' data reported to FAOSTAT shown in Fig. 3 for the 2010s, due to differences in food group coverage and how regions are defined. From then onwards, model projections show faster improvement in regions with lower HDBI levels and slower improvements elsewhere. The investment scenario represents supply-side enhancements that increase supply of some food groups (starchy staples, animal-source foods, pulses and oilseeds) and increase resource use efficiency and hence reduce consumer prices for those food groups. Although these investments do not directly target fruits and vegetables, they indirectly influence the supply of these foods via income and demand effects. Within the increased investment scenario, the largest effect comes from the broad category of international R&D that leads to genetic improvement with accompanying changes in agronomic or veterinary management. This category of innovation has by far the most leverage, raising the productivity of all land, labor and other inputs used for each product, whereas the other forms of investment address important but much more limited opportunities for cost reduction or resource use. The availability of foods needed for healthy diets rises most quickly in South Asia and more slowly in Sub-Saharan Africa. In both of those regions, higher international R&D investments would accelerate progress by about a decade's worth of otherwise gradual change. However, by 2050, the average national HDBI of 0.74 with, and 0.72 without new investments would still fall short of full alignment with food group recommendations (see Table S4). This primarily reflects significant gaps for legumes, nuts and seeds across all world regions, as well as gaps for fruits and vegetables in Sub-Saharan Africa, fruits and vegetables in South Asia, and vegetables in Latin America & Caribbean.

These estimates reflect relatively pessimistic socioeconomic assumptions under the SSP3 shared socioeconomic pathway and the RCP8.5 climate-change scenario. Assuming new investments and the RCP8.5 climate-change scenario, national average HDBI would be about 0.79 by 2050 under SSP1, compared with 0.76 under SSP2 and 0.74 under SSP3 (see Fig. S3). In contrast, assuming no new investments and the RCP8.5 climate-change scenario, national average HDBI would be about 0.78 by 2050 under SSP1, compared with 0.75 under SSP2 and 0.72 under SSP3. In general, the projected increase in HDBI due to the modeled investment changes is smaller than the projected increase in HDBI that would occur under optimistic socioeconomic conditions (SSP1) compared to more pessimistic conditions (SSP3).

## 4 Discussion and conclusion

To meet global targets, Sub-Saharan Africa needs greater availability of all food groups except starchy staples; South Asia needs greater availability of vegetables, fruit, and animal source foods; East Asia and the Pacific need more fruit; and all regions of the world needs more legumes, nuts and seeds. These results are consistent with the nutrient-based findings highlighted previously in Section 1. For example, global calcium shortfalls are likely driven by the low availability of dairy and seafood compared to other animal-source foods and by shortfalls of vegetables, including calcium-rich dark green leafy vegetables. Regional averages also obscure cases where disparities exist between or within countries; for example, East Asia and the Pacific is sufficient for vegetables only due to the outsize role of China, while nearby countries still face deep deficits. At current retail prices, even the least expensive locally available items in each HDB food group cost more per day than the available income for nearly 3 billion people worldwide (FAO et al., 2024; World Bank, 2024). Accelerated investments in agriculture help close the gap somewhat faster than “business as usual,” despite both worsening environmental conditions due to climate change and relatively pessimistic socioeconomic conditions under SSP3 assumptions, especially in South Asia and Sub-Saharan Africa. Our projections, however, show that investing in supply-side measures to lower prices for some food groups would bring food supplies only part way toward the quantities needed for healthy diets. Additional investments that are explicitly aimed at increasing supply and demand for recommended foods will also be necessary to achieve this goal.

There are many factors that have driven food system advancements around the world since 1961, and that simultaneously explain some of the shortfalls that persist for key nutritious food groups, especially in Sub-Saharan Africa and South Asia. Government investments in agriculture commonly known as the Green Revolution brought innovations in crop and livestock genetics, irrigation and fertilizer technologies, cultivation equipment, and other aspects of food production systems. These innovations boosted productivity and helped to expand the availability of staple grains for human and animal consumption. Higher yields and the other labor-saving effects of new agricultural technologies led to higher incomes among farming households, which led to greater demand for aspirational foods such as meat, dairy, oils, fruits, and vegetables. However, these types of agricultural investments have never been fully implemented in Sub-Saharan Africa, and as a result the productivity growth that occurred in Asia and Latin America has not yet developed there in the same way (Pingali et al., 2023). Furthermore, a new tradition of agricultural investments will have to account for and respond to environmental degradation and climate change in ways that Green Revolution technologies did not (Barrett et al., 2023).

Each country and region of the world has its own story of food system development and transformation. In East Asia & Pacific, much of the exponential growth in nutrient-rich fruits, vegetables, and animal-source foods is driven by China, where the reforms of the 1970s and 1980s led to unprecedented economic growth and where the subsequent dietary transition toward fruits, vegetables, and animal source foods far outpaced the similar shift taking place elsewhere in East and South Asia (Lele and Goswami, 2020; Pingali and Abraham, 2022). However, the role of income growth is a common theme across all countries and cultural histories in the form of Bennett's Law, which finds that as incomes rise, demand for starchy staples and plant sources of protein declines as consumers switch to more expensive animal source foods (Masters et al., 2022). Our analysis of past food supply trends aligns with this principle; while per capita global and regional supplies for starchy staples and legumes, nuts and seeds have remained essentially unchanged since the 1960s, consumer demand for meat, milk, vegetable oils, vegetables, and fruits has risen substantially. This pattern suggests that ongoing income growth around the world will lead to continued expansion in the availability of these five broad categories of foods—an expansion that will only be equitably distributed if income growth is also equitable. Furthermore, the IMPACT model projections show that limited demand for legumes, nuts and seeds is a major barrier to full food system alignment with dietary guidelines. Although consumption of aspirational food groups such as fruits, vegetables, and animal-source foods may arise from income growth and agricultural R&D, additional efforts will be necessary to create demand for plant sources of protein.

This analysis is subject to several limitations. There are well-known limitations to using FBS data, which have been extensively documented elsewhere (Thar et al., 2020; Cafiero, 2013). These include variation and error in country-level reporting, and the exclusion of foods from informal trade, foraging, and other unofficial sources, which may result in underreporting of totals for some foods. A change in the FAO methodology for balancing food supply data across input categories results in a discontinuity between data before and after 2010. We perform no adjustments to account for this discontinuity but instead present all data as published in FAOSTAT. Also, our approach concerns only the availability of raw ingredients and allows for the analysis of food system trends over time, as shifts in domestic production, trade policy, and research and development lead to increased or decreased availability of each food group. We do not account for consumer-level food waste. Although food loss and waste estimates from FAO are commonly used in nutrition research (FAO, 2011), more up-to-date estimates for avoidable food waste by national income level are not available. Instead, our results reflect the potential availability of human food, a portion of which consumers may choose to discard or feed to pets, especially in high-income countries where rates of avoidable food waste are higher (Gatto and Chepeliev, 2024).

One important limitation of this work is that we do not discuss the availability of unhealthy foods in global food systems. The FBS does not address whether primary ingredients that belong to the required HDB food groups are transformed into unhealthy foods that are not required in dietary guidelines through combination with additional ingredients, processing or contamination. Similarly, the HDBI approach does not include a penalty for excess availability of any food groups. Future work could expand the HDBI approach to account for excess energy intake and consumption of unhealthy foods, and would also address a different set of policy concerns around the health consequences of diets that overshoot food-based recommendations.

We also do not account for inequities in access or distribution, including reallocation of food between countries through international trade. This paper does not seek to assess access, which is discussed at length in the SOFI reports, but seeks to unpack availability as one of its upstream determinants. Food supplies provide information on the theoretical ability of all people to access adequate food given sufficient economic resources. Yearly national data do not allow for estimates of subnational or temporal variation in food availability. This obscures the important observation that even in places where national food supplies are in full alignment with dietary guidelines, populations in given places and at given times may not in fact have physical or economic access to healthy diets. For food groups where global availability meets or exceeds HDB reference quantities while regional availability falls short, international trade could in principle redistribute that excess to create a more equitable food supply. This would not be sufficient to correct global shortfalls in fruits, vegetables, and legumes, nuts, and seeds. Realigning international demand with FBDGs in this way would also require interventions to reduce demand in places with excess availability, so that demand in places where food groups fall short could be met or increased.

Does the world produce enough food? In calories, yes. In all foods needed for a healthy diet, no. Past improvements and projected future investments have closed some but not all the global shortfall in supply and demand of all food groups needed for a healthy diet, leaving large gaps for several food groups in specific regions of the world. Notably, past and projected investments leave significant shortfalls that are highly concentrated in specific food groups and geographic regions. Investments in “food” or “agriculture” need to go beyond the set of crops that have typically received the vast majority of investment – staple grains, starchy roots and tubers, and oil crops (Herforth et al., 2012; Pingali, 2015) – and focus on the foods that are the most in need: fruits, vegetables, legumes, nuts and seeds. Targeted investments would be needed to raise both demand and supply for locally-adapted foods.

These reconsidered supply side investments may also need to be accompanied by other policy levers to counteract income-driven trends toward greater consumption of animal-source foods and oils and fats. Although people with low incomes are frequently unable to afford the foods required for a healthy diet, many high-income people choose to consume less-healthy diets even though healthy foods are available and affordable. Demand creation through higher incomes or social assistance as well as innovation may be needed to make under-consumed foods more attractive to consumers, alongside innovation to improve supply response, and open trade needed to keep prices low. Shifting consumption patterns toward healthy diets will require not only demand-side policy, but coordinated investment in R&D including a new focus on food groups for which there are large and persistent shortfalls in availability.

## CRediT authorship contribution statement

**Leah Costlow:** Writing – review & editing, Writing – original draft, Visualization, Formal analysis, Data curation. **Anna Herforth:** Writing – review & editing, Methodology, Conceptualization. **Timothy B. Sulser:** Writing – review & editing, Resources. **Nicola Cenacchi:** Writing – review & editing, Resources. **William A. Masters:** Writing – review & editing, Methodology, Conceptualization.

## Code availability

All data analysis and visualizations were conducted in RStudio (version 2024.04.1 + 748). All R scripts will be available at https://osf.io/kjy4z/.

## Declaration of competing interest

The authors declare that they have no known competing financial interests or personal relationships that could have appeared to influence the work reported in this paper.

## Data Availability

Data will be made available on request.
