# Supplementary material for: Global analysis reveals persistent shortfalls and regional differences in availability of foods needed for health
Source: Glob Food Sec. 2025 Mar;44:100825. doi: 10.1016/j.gfs.2024.100825 (PMC11908977; doi:10.1016/j.gfs.2024.100825)
Supplement: Multimedia component 1 [file mmc1.docx]

**Supplementary Information**

**Figure S1. Projected HDBI scores showing adequacy of global and regional food supplies to meet healthy diet basket amounts, 2010-2050.**

**
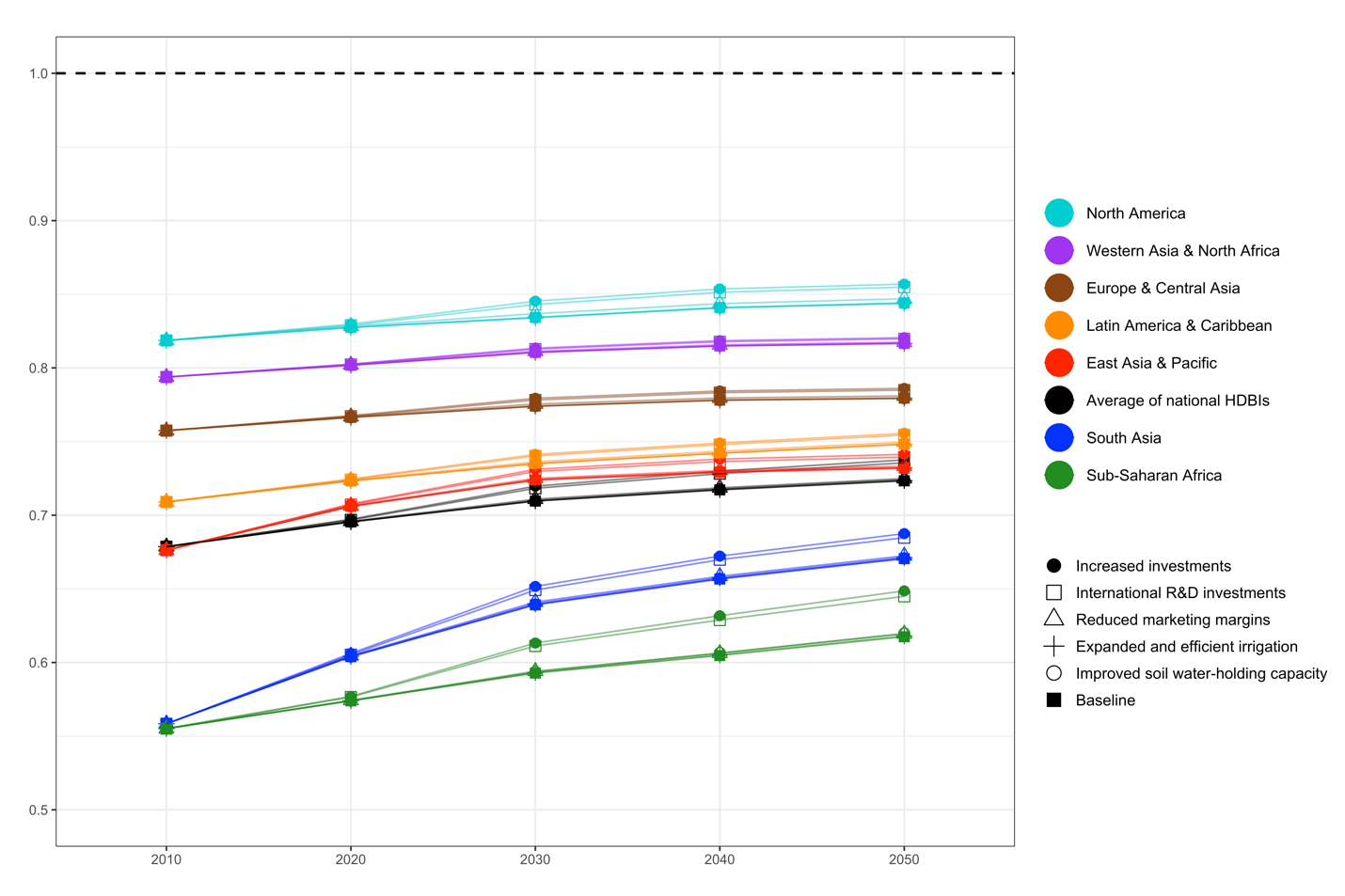
**

Note: Points show means of all countries’ average proportion of target availability for the six HDB food groups, by geographic region and decade. Solid squares show business-as-usual investment levels, solid circles show outcomes from a higher level of investment in agricultural R&D complemented by improvements in water management and infrastructure, while the four other symbols shown in the legend show partial packages giving intermediate results. All outcomes are IMPACT model estimates given the SSP3 shared socioeconomic pathway and RCP8.5 climate-change scenario, using IMPACT to compute annual percentage changes in daily energy per capita of each commodity available for food use from the base year of observed quantities in 2010. Country inclusion described in the methods section and in Supplementary Table 5.

**Figure S2. Food supplies in East Asia & Pacific and mainland China relative to Healthy Diet Basket targets, 1961-2022.**

**
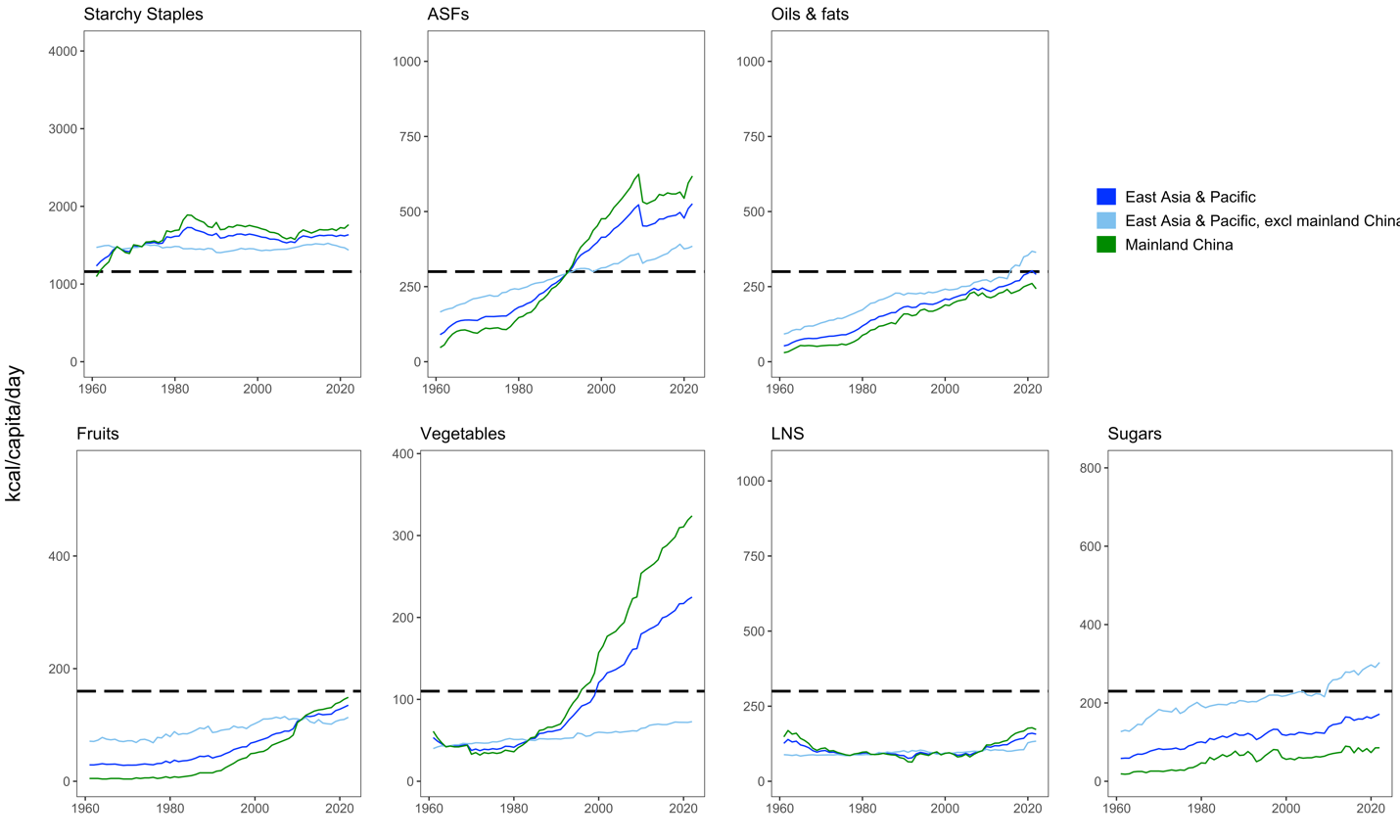
**

Note: Data shown are worldwide totals for daily per capita kilocalories available as food, computed by the authors from national data in FAOSTAT. Plots are scaled to reference intake values. Black dashed lines indicate Healthy Diet Basket targets used to measure access to a balanced diet as recommended in national dietary guidelines. Sugar availability is shown in reference to the WHO guideline that sugar intake be limited to 10% or less of total daily energy (WHO 2015).

**Figure S3. Projected average of national HDBIs with status quo investments, by Shared Socioeconomic Pathway.**

**
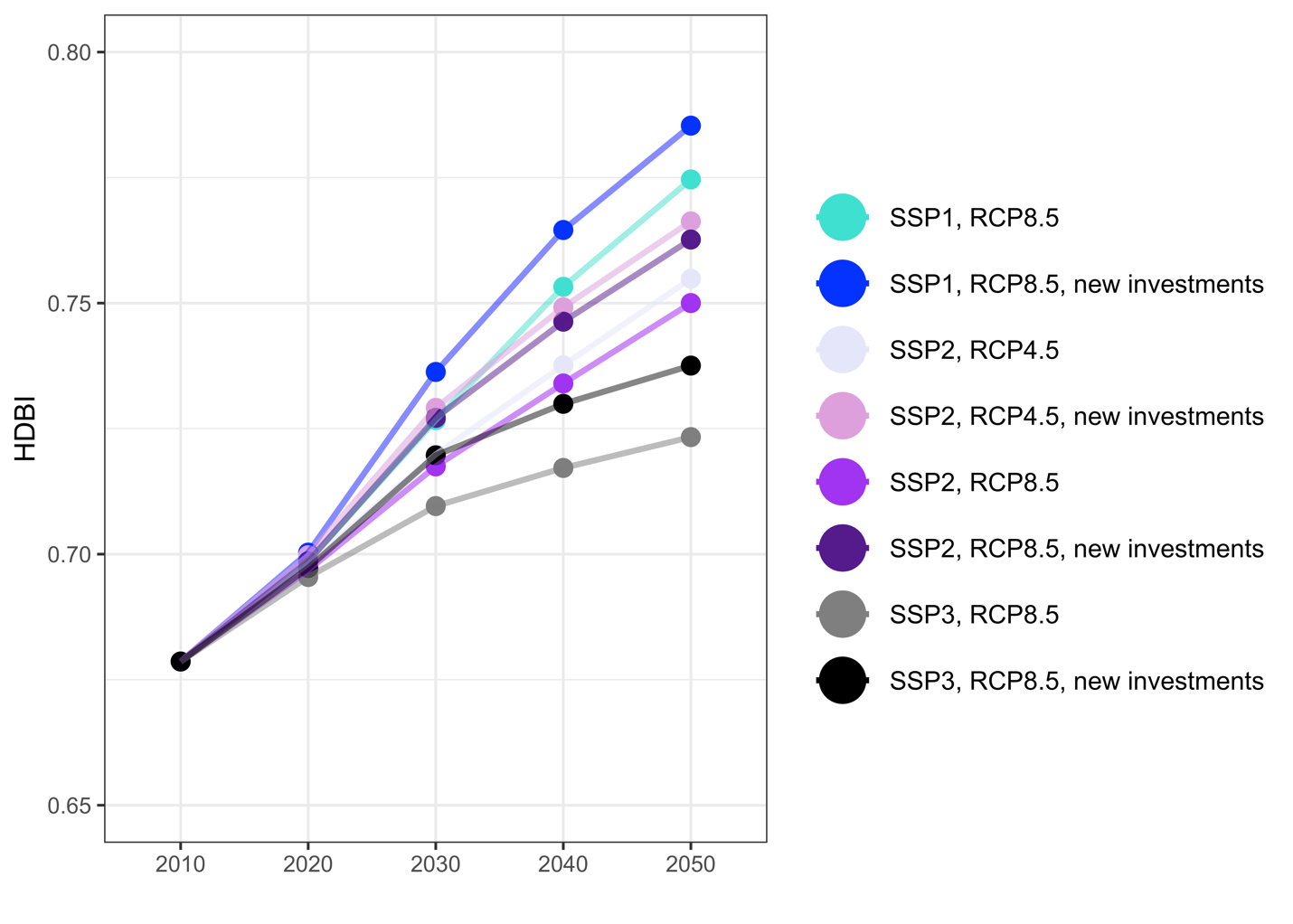
**

Note: Points show means of all countries’ average proportion of target availability for the six Healthy Diet Basket (HDB) food groups by decade. Scenarios in dark gray (SSP3, RCP8.5) and black (SSP3, RCP8.5, new investments) correspond to the lines labeled “Average of national HDBIs” in Figure 4. Vertical axis is restricted for higher resolution. Scenarios include moderate degrees of climate change (RCP4.5) and climate change with continued growth in emissions (RCP8.5).

**Table S1. FAO commodity regroupings used in Figure 1.**

| FAO Commodity Code | FAO Commodity Name | New Grouping |
| --- | --- | --- |
| 2612 | Lemons, Limes and products | Other fruits |
| 2613 | Grapefruit and products | Other fruits |
| 2614 | Citrus, Other | Other fruits |
| 2618 | Pineapples and products | Other fruits |
| 2625 | Fruits, other | Other fruits |
| 2605 | Other vegetables | Other vegetables |
| 2775 | Aquatic plants | Other vegetables |
| 2768 | Meat, aquatic mammals | Other meats |
| 2736 | Edible offals | Other meats |
| 2735 | Meat, other | Other meats |
| 2516 | Oats | Other cereals |
| 2515 | Rye and products | Other cereals |
| 2513 | Barley and products | Other cereals |
| 2520 | Cereals, Other | Other cereals |
| 2549 | Other pulses and products | Other pulses and seeds |
| 2557 | Sunflower seeds | Other pulses and seeds |
| 2561 | Sesame seeds | Other pulses and seeds |
| 2535 | Yams | Other roots |
| 2534 | Roots, other | Other roots |
| 2769 | Aquatic Animals, Others | Other seafood |
| 2767 | Molluscs, Other | Other seafood |
| 2766 | Cephalopods | Other seafood |
| 2765 | Crustaceans | Other seafood |
| 2764 | Marine Fish, Other | Marine fish |
| 2762 | Demersal fish | Marine fish |
| 2763 | Pelagic fish | Marine fish |
| 2782 | Fish, Liver Oil | Other oils and oilcrops |
| 2781 | Fish, Body Oil | Other oils and oilcrops |
| 2579 | Sesameseed Oil | Other oils and oilcrops |
| 2581 | Ricebran Oil | Other oils and oilcrops |
| 2576 | Palmkernal Oil | Other oils and oilcrops |
| 2582 | Maize Germ Oil | Other oils and oilcrops |
| 2586 | Oilcrops Oil, Other | Other oils and oilcrops |
| 2562 | Palm kernels | Other oils and oilcrops |
| 2558 | Rape and mustardseed | Other oils and oilcrops |
| 2559 | Cottonseed | Other oils and oilcrops |
| 2743 | Cream | Other oils and oilcrops |
| 2570 | Oilcrops, Other | Other oils and oilcrops |
| 2543 | Sweeteners, Other | Other sweeteners |
| 2745 | Honey | Other sweeteners |
| 2536 | Sugar cane | Other sweeteners |
| 2541 | Non-centrifugal sugar | Other sweeteners |
| 2537 | Sugar beet | Other sweeteners |

| Table S2. National HDBI and food supply as percentage of reference intake (first and last year available). | | | | | | | | | |
| --- | --- | --- | --- | --- | --- | --- | --- | --- | --- |
| Country | Year | Animal-Source Foods | Fruits | Legumes Nuts & Seeds | Oils & Fats | Starchy Staples | Sugars | Vegetables | HDBI |
| Afghanistan | 1961 | 0.663 | 0.369 | 0.117 | 0.243 | 2.203 | 0.222 | 0.2 | 0.432 |
|  | 2022 | 0.421 | 0.352 | 0.21 | 0.822 | 1.395 | 0.37 | 0.364 | 0.528 |
| Albania | 1961 | 0.957 | 0.575 | 0.173 | 0.487 | 1.268 | 0.478 | 0.373 | 0.594 |
|  | 2022 | 3.164 | 2.004 | 0.367 | 1.096 | 0.889 | 1.067 | 2.286 | 0.876 |
| Algeria | 1961 | 0.537 | 0.369 | 0.103 | 0.43 | 0.893 | 0.696 | 0.191 | 0.42 |
|  | 2022 | 1.27 | 1.39 | 0.274 | 1.832 | 1.498 | 1.263 | 1.477 | 0.879 |
| Angola | 1961 | 0.31 | 0.412 | 0.337 | 0.41 | 1.083 | 0.37 | 0.164 | 0.439 |
|  | 2022 | 0.58 | 0.828 | 0.358 | 0.862 | 1.334 | 0.417 | 0.331 | 0.66 |
| Antigua and Barbuda | 1961 | 1.153 | 0.75 | 0.047 | 0.837 | 0.779 | 1.535 | 0.055 | 0.578 |
|  | 2022 | 2.224 | 1.325 | 0.132 | 1.284 | 0.589 | 0.934 | 0.5 | 0.704 |
| Argentina | 1961 | 2.987 | 0.531 | 0.093 | 0.813 | 1.047 | 1.661 | 0.509 | 0.658 |
|  | 2022 | 3.054 | 0.543 | 0.379 | 1.815 | 0.972 | 1.614 | 0.513 | 0.735 |
| Armenia | 1992 | 1.087 | 0.525 | 0 | 0.13 | 1.213 | 0.948 | 0.691 | 0.724 |
|  | 2022 | 2.662 | 1.023 | 0.236 | 1.43 | 1.068 | 1.332 | 1.359 | 0.873 |
| Australia | 1961 | 3.2 | 0.675 | 0.12 | 1.22 | 0.762 | 2.43 | 0.364 | 0.653 |
|  | 2022 | 2.916 | 0.703 | 0.609 | 2.434 | 0.658 | 1.838 | 0.698 | 0.778 |
| Austria | 1961 | 2.253 | 1.1 | 0.09 | 1.81 | 0.978 | 1.704 | 0.373 | 0.74 |
|  | 2022 | 2.654 | 0.909 | 0.428 | 3.515 | 0.849 | 1.661 | 0.738 | 0.821 |
| Azerbaijan | 1992 | 0.967 | 0.731 | 0.08 | 0.32 | 1.343 | 0.578 | 0.282 | 0.563 |
|  | 2022 | 1.799 | 0.824 | 0.271 | 1.408 | 1.37 | 1.277 | 1.164 | 0.849 |
| Bahamas | 1961 | 1.673 | 0.606 | 0.163 | 0.72 | 0.825 | 1.583 | 0.864 | 0.696 |
|  | 2022 | 2.586 | 1.418 | 0.154 | 1.149 | 0.472 | 1.323 | 0.892 | 0.753 |
| Bahrain | 2019 | 2.288 | 1.048 | 0.449 | 1.859 | 1.019 | 2.143 | 0.947 | 0.899 |
|  | 2022 | 2.124 | 1.056 | 0.486 | 2.06 | 1.023 | 2.09 | 0.961 | 0.908 |
| Bangladesh | 1961 | 0.183 | 0.181 | 0.17 | 0.223 | 1.571 | 0.383 | 0.109 | 0.311 |
|  | 2022 | 0.737 | 0.268 | 0.254 | 0.647 | 1.61 | 0.339 | 0.433 | 0.556 |
| Barbados | 1961 | 1.633 | 0.381 | 0.3 | 0.553 | 0.966 | 2.204 | 0.1 | 0.55 |
|  | 2022 | 2.265 | 0.8 | 0.414 | 1.312 | 0.86 | 2.35 | 0.661 | 0.789 |
| Belarus | 1992 | 2.513 | 0.294 | 0.017 | 0.947 | 1.276 | 1.461 | 0.4 | 0.61 |
|  | 2022 | 2.652 | 0.596 | 0.154 | 1.854 | 0.94 | 1.564 | 1.033 | 0.782 |
| Belgium | 2000 | 2.497 | 0.581 | 0.24 | 3.15 | 0.863 | 2.27 | 1.064 | 0.781 |
|  | 2022 | 3.103 | 0.758 | 0.329 | 3.659 | 0.752 | 2.168 | 0.978 | 0.803 |
| Belgium-Luxembourg | 1961 | 1.96 | 0.5 | 0.1 | 2.193 | 0.9 | 1.161 | 0.518 | 0.67 |
|  | 1999 | 2.473 | 0.819 | 0.193 | 3.143 | 0.826 | 2.139 | 1.2 | 0.806 |
| Belize | 1961 | 1.28 | 0.662 | 0.303 | 0.84 | 0.822 | 1.13 | 0.118 | 0.624 |
|  | 2022 | 1.389 | 0.176 | 0.684 | 1.632 | 0.925 | 1.544 | 0.288 | 0.679 |
| Benin | 1961 | 0.207 | 0.369 | 0.367 | 0.527 | 1.122 | 0.104 | 0.1 | 0.428 |
|  | 2022 | 0.446 | 0.268 | 0.89 | 1.057 | 1.489 | 0.381 | 0.408 | 0.669 |
| Bermuda | 1961 | 2.72 | 0.775 | 0.127 | 1.297 | 0.525 | 1.73 | 0.627 | 0.676 |
|  | 2009 | 2.557 | 0.525 | 0.137 | 1.347 | 0.57 | 1.526 | 0.709 | 0.657 |
| Bhutan | 2019 | 0.833 | 0.225 | 0.173 | 1.582 | 1.767 | 0.645 | 0.412 | 0.607 |
|  | 2022 | 0.775 | 0.281 | 0.229 | 1.494 | 1.8 | 0.634 | 0.396 | 0.614 |
| Bolivia | 1961 | 0.617 | 0.475 | 0.113 | 0.473 | 0.893 | 0.848 | 0.464 | 0.506 |
|  | 2022 | 1.507 | 0.427 | 0.305 | 0.51 | 1.024 | 1.163 | 0.342 | 0.597 |
| Bosnia and Herzegovina | 1992 | 0.893 | 0.188 | 0.197 | 0.127 | 1.323 | 0.422 | 0.736 | 0.523 |
|  | 2022 | 1.988 | 1.554 | 0.362 | 0.88 | 1.145 | 1.258 | 1.53 | 0.874 |
| Botswana | 1961 | 0.917 | 0.081 | 0.77 | 0.26 | 0.975 | 0.717 | 0.091 | 0.516 |
|  | 2022 | 1.03 | 0.298 | 0.158 | 0.977 | 1.143 | 1.132 | 0.269 | 0.617 |
| Brazil | 1961 | 0.793 | 0.631 | 0.773 | 0.46 | 0.9 | 1.783 | 0.155 | 0.619 |
|  | 2022 | 2.62 | 0.97 | 0.405 | 2.326 | 0.881 | 1.648 | 0.335 | 0.765 |
| Brunei Darussalam | 1961 | 0.743 | 0.35 | 0.167 | 0.53 | 0.922 | 1.474 | 0.191 | 0.484 |
|  | 2009 | 1.79 | 0.544 | 0.283 | 0.977 | 1.141 | 1.691 | 0.564 | 0.728 |
| Bulgaria | 1961 | 1.12 | 0.738 | 0.223 | 1.133 | 1.653 | 0.861 | 0.573 | 0.756 |
|  | 2022 | 2.031 | 0.556 | 0.314 | 2.265 | 0.888 | 1.39 | 0.592 | 0.725 |
| Burkina Faso | 1961 | 0.21 | 0.038 | 0.813 | 0.1 | 0.701 | 0.074 | 0.136 | 0.333 |
|  | 2022 | 0.689 | 0.439 | 1.843 | 0.352 | 1.332 | 0.238 | 0.389 | 0.645 |
| Burundi | 2010 | 0.098 | 1.698 | 0.765 | 0.212 | 0.668 | 0.14 | 0.279 | 0.504 |
|  | 2022 | 0.122 | 0.822 | 0.894 | 0.223 | 0.967 | 0.265 | 0.236 | 0.544 |
| Cabo Verde | 1961 | 0.167 | 0.588 | 0.4 | 0.153 | 0.981 | 0.626 | 0.045 | 0.389 |
|  | 2022 | 1.202 | 0.47 | 0.377 | 0.976 | 1.199 | 0.911 | 0.481 | 0.717 |
| Cambodia | 1961 | 0.173 | 0.344 | 0.127 | 0.083 | 1.37 | 0.465 | 0.3 | 0.338 |
|  | 2022 | 0.616 | 0.119 | 0.193 | 0.483 | 1.784 | 0.425 | 0.234 | 0.441 |
| Cameroon | 1961 | 0.29 | 0.1 | 0.53 | 0.26 | 1.338 | 0.096 | 0.218 | 0.4 |
|  | 2022 | 0.457 | 0.38 | 0.882 | 0.908 | 1.554 | 0.583 | 0.647 | 0.712 |
| Canada | 1961 | 2.45 | 0.637 | 0.213 | 1.65 | 0.654 | 2.048 | 0.536 | 0.674 |
|  | 2022 | 2.332 | 0.864 | 0.665 | 3.02 | 0.856 | 1.887 | 0.751 | 0.856 |
| Central African Republic | 1961 | 0.25 | 0.3 | 0.143 | 0.96 | 1.499 | 0.117 | 0.1 | 0.459 |
|  | 2022 | 0.736 | 0.423 | 1.412 | 1.178 | 0.847 | 0.331 | 0.132 | 0.69 |
| Chad | 1961 | 0.487 | 0.288 | 1.21 | 0.407 | 1.31 | 0.213 | 0.073 | 0.542 |
|  | 2022 | 1.157 | 0.082 | 1.041 | 0.569 | 1.06 | 0.422 | 0.04 | 0.615 |
| Chile | 1961 | 1.14 | 0.306 | 0.19 | 0.683 | 1.167 | 1.165 | 0.782 | 0.66 |
|  | 2022 | 2.129 | 0.481 | 0.231 | 1.196 | 1.046 | 1.998 | 0.604 | 0.719 |
| China, Hong Kong SAR | 1961 | 1.087 | 0.238 | 0.507 | 1.18 | 1.178 | 0.891 | 1.082 | 0.791 |
|  | 2022 | 2.908 | 0.622 | 0.361 | 1.415 | 0.708 | 1.204 | 1.377 | 0.782 |
| China, Macao SAR | 1961 | 0.767 | 0.162 | 0.443 | 1 | 1.438 | 1 | 0.418 | 0.632 |
|  | 2022 | 2.501 | 0.507 | 0.242 | 1.617 | 0.791 | 1.071 | 0.884 | 0.737 |
| China, mainland | 1961 | 0.157 | 0.031 | 0.493 | 0.1 | 0.945 | 0.083 | 0.555 | 0.38 |
|  | 2022 | 2.06 | 0.932 | 0.575 | 0.81 | 1.521 | 0.372 | 2.946 | 0.886 |
| China, Taiwan Province of | 1961 | 0.703 | 0.15 | 0.35 | 0.33 | 1.547 | 0.622 | 0.555 | 0.515 |
|  | 2022 | 2.034 | 0.823 | 0.616 | 2.272 | 0.759 | 1.118 | 0.934 | 0.855 |
| Colombia | 1961 | 1.073 | 0.244 | 0.16 | 0.387 | 0.882 | 2.452 | 0.155 | 0.471 |
|  | 2022 | 1.942 | 1.093 | 0.22 | 1.433 | 0.971 | 2.573 | 0.363 | 0.759 |
| Comoros | 2010 | 0.558 | 1.788 | 0.637 | 1.009 | 1.145 | 0.465 | 0.093 | 0.715 |
|  | 2022 | 0.619 | 2.315 | 0.617 | 0.428 | 1.157 | 0.634 | 0.198 | 0.644 |
| Congo | 1961 | 0.357 | 0.238 | 0.33 | 0.563 | 1.384 | 0.083 | 0.082 | 0.428 |
|  | 2022 | 0.914 | 0.294 | 0.164 | 0.937 | 1.107 | 0.614 | 0.118 | 0.571 |
| Costa Rica | 1961 | 0.783 | 0.6 | 0.31 | 0.703 | 0.734 | 1.896 | 0.109 | 0.54 |
|  | 2022 | 2.255 | 0.615 | 0.408 | 1.634 | 0.799 | 1.944 | 0.341 | 0.694 |
| Cote d'Ivoire | 1961 | 0.423 | 0.075 | 0.5 | 0.37 | 1.523 | 0.343 | 0.273 | 0.44 |
|  | 2022 | 0.434 | 0.079 | 0.245 | 0.929 | 1.883 | 0.546 | 0.302 | 0.498 |
| Croatia | 1992 | 1.543 | 0.481 | 0.12 | 0.96 | 0.728 | 1.704 | 0.355 | 0.607 |
|  | 2022 | 2.638 | 0.737 | 0.235 | 2.04 | 0.767 | 1.861 | 1.371 | 0.79 |
| Cuba | 1961 | 1 | 0.244 | 0.473 | 0.807 | 0.74 | 2.074 | 0.209 | 0.579 |
|  | 2022 | 1.559 | 0.667 | 0.447 | 0.988 | 1.356 | 2.06 | 0.754 | 0.809 |
| Cyprus | 1961 | 0.967 | 1.225 | 0.64 | 1.22 | 0.923 | 0.948 | 0.5 | 0.838 |
|  | 2022 | 2.411 | 0.587 | 0.327 | 1.48 | 0.949 | 1.108 | 0.625 | 0.748 |
| Czechia | 1993 | 2.49 | 0.506 | 0.13 | 1.893 | 0.772 | 1.687 | 0.427 | 0.639 |
|  | 2022 | 2.4 | 0.639 | 0.228 | 3.122 | 0.676 | 1.544 | 0.562 | 0.684 |
| Czechoslovakia | 1961 | 2.03 | 0.419 | 0.093 | 1.717 | 1.214 | 1.922 | 0.4 | 0.652 |
|  | 1992 | 2.19 | 0.469 | 0.093 | 1.89 | 0.928 | 1.965 | 0.364 | 0.642 |
| Democratic Republic of the Congo | 2010 | 0.114 | 0.141 | 0.273 | 0.444 | 1.637 | 0.156 | 0.061 | 0.339 |
|  | 2022 | 0.119 | 0.153 | 0.23 | 0.358 | 1.601 | 0.149 | 0.045 | 0.317 |
| Denmark | 1961 | 2.08 | 0.469 | 0.023 | 2.667 | 0.872 | 2.165 | 0.245 | 0.602 |
|  | 2022 | 3.191 | 0.789 | 0.265 | 1.928 | 0.835 | 2.154 | 0.693 | 0.764 |
| Djibouti | 1961 | 0.697 | 0 | 0.093 | 0.283 | 0.765 | 1.517 | 0.055 | 0.482 |
|  | 2022 | 0.548 | 0.299 | 0.689 | 1.247 | 1.261 | 1.573 | 0.453 | 0.665 |
| Dominica | 1961 | 0.733 | 1.1 | 0.043 | 0.423 | 0.624 | 1.509 | 0.336 | 0.527 |
|  | 2022 | 2.282 | 2.644 | 0.2 | 0.737 | 0.74 | 1.172 | 0.817 | 0.749 |
| Dominican Republic | 1961 | 0.607 | 1.225 | 0.387 | 0.387 | 0.72 | 0.987 | 0.109 | 0.535 |
|  | 2022 | 1.71 | 3.236 | 0.292 | 1.492 | 0.891 | 1.817 | 0.821 | 0.834 |
| Ecuador | 1961 | 0.93 | 1.425 | 0.417 | 0.637 | 0.745 | 1.117 | 0.355 | 0.68 |
|  | 2022 | 1.553 | 0.263 | 0.111 | 1.951 | 0.902 | 1.236 | 0.17 | 0.574 |
| Egypt | 1961 | 0.337 | 0.456 | 0.267 | 0.523 | 1.247 | 0.6 | 0.655 | 0.54 |
|  | 2022 | 0.944 | 1.033 | 0.145 | 0.704 | 1.742 | 1.108 | 0.859 | 0.775 |
| El Salvador | 1961 | 0.57 | 0.288 | 0.23 | 0.417 | 0.785 | 0.909 | 0.155 | 0.407 |
|  | 2022 | 1.415 | 0.642 | 0.581 | 0.834 | 1.198 | 1.441 | 0.549 | 0.768 |
| Estonia | 1992 | 3.067 | 0.2 | 0.01 | 0.58 | 0.888 | 0.817 | 0.318 | 0.499 |
|  | 2022 | 3.316 | 0.734 | 0.309 | 1.877 | 0.618 | 1.58 | 0.593 | 0.709 |
| Eswatini | 1961 | 0.94 | 0.181 | 0.433 | 0.187 | 1.16 | 1.209 | 0.082 | 0.471 |
|  | 2022 | 0.809 | 0.483 | 0.206 | 0.787 | 1.224 | 1.437 | 0.197 | 0.58 |
| Ethiopia | 1993 | 0.227 | 0.025 | 0.317 | 0.143 | 1.06 | 0.157 | 0.064 | 0.296 |
|  | 2022 | 0.339 | 0.143 | 0.783 | 0.619 | 1.534 | 0.336 | 0.123 | 0.501 |
| Ethiopia PDR | 1961 | 0.487 | 0.019 | 0.603 | 0.24 | 1.173 | 0.096 | 0.055 | 0.401 |
|  | 1992 | 0.273 | 0.025 | 0.49 | 0.273 | 1.012 | 0.13 | 0.055 | 0.353 |
| Fiji | 1961 | 0.623 | 1.556 | 0.123 | 0.523 | 1.409 | 1.235 | 0.091 | 0.56 |
|  | 2022 | 1.275 | 0.769 | 0.29 | 1.412 | 1.118 | 1.848 | 0.731 | 0.798 |
| Finland | 1961 | 3.033 | 0.356 | 0.047 | 1.567 | 1.105 | 1.913 | 0.127 | 0.588 |
|  | 2022 | 3.399 | 0.645 | 0.293 | 1.233 | 0.991 | 1.449 | 0.606 | 0.756 |
| France | 1961 | 2.737 | 0.45 | 0.147 | 1.263 | 1.002 | 1.287 | 0.936 | 0.756 |
|  | 2022 | 3.035 | 0.832 | 0.255 | 2.353 | 0.867 | 1.616 | 0.697 | 0.775 |
| French Polynesia | 1961 | 1.237 | 0.887 | 0.12 | 0.71 | 1.181 | 1.517 | 0.3 | 0.67 |
|  | 2022 | 2.33 | 1.032 | 0.187 | 1.508 | 0.86 | 1.062 | 0.369 | 0.736 |
| Gabon | 1961 | 0.707 | 0.262 | 0.123 | 0.243 | 1.228 | 0.113 | 0.245 | 0.43 |
|  | 2022 | 1.3 | 0.186 | 0.585 | 0.255 | 1.353 | 0.668 | 0.232 | 0.543 |
| Gambia | 1961 | 0.337 | 0.044 | 0.6 | 0.803 | 1.191 | 0.487 | 0.073 | 0.476 |
|  | 2022 | 0.62 | 0.041 | 0.423 | 0.899 | 1.1 | 2.288 | 0.162 | 0.524 |
| Georgia | 1992 | 0.893 | 0.594 | 0.11 | 0.18 | 1.068 | 0.5 | 0.255 | 0.505 |
|  | 2022 | 1.912 | 0.577 | 0.167 | 0.988 | 1.274 | 1.676 | 0.435 | 0.695 |
| Germany | 1961 | 1.927 | 0.681 | 0.123 | 2.003 | 0.835 | 1.465 | 0.282 | 0.654 |
|  | 2022 | 2.916 | 0.804 | 0.353 | 2.893 | 0.733 | 1.987 | 0.681 | 0.762 |
| Ghana | 1961 | 0.353 | 0.537 | 0.147 | 0.417 | 1.302 | 0.435 | 0.173 | 0.438 |
|  | 2022 | 0.494 | 0.354 | 0.475 | 0.699 | 2.011 | 0.539 | 0.194 | 0.536 |
| Greece | 1961 | 1.123 | 1.25 | 0.423 | 1.527 | 1.196 | 0.648 | 0.618 | 0.84 |
|  | 2022 | 2.668 | 1.338 | 0.514 | 2.235 | 0.775 | 1.497 | 0.737 | 0.838 |
| Grenada | 1961 | 0.633 | 1.819 | 0.237 | 0.527 | 0.58 | 1.187 | 0.155 | 0.522 |
|  | 2022 | 2.21 | 1.245 | 0.294 | 1.184 | 0.487 | 1.363 | 0.465 | 0.708 |
| Guatemala | 1961 | 0.463 | 0.288 | 0.307 | 0.247 | 1.038 | 0.909 | 0.164 | 0.411 |
|  | 2022 | 1.04 | 1.021 | 0.422 | 0.901 | 1.069 | 2.458 | 0.517 | 0.807 |
| Guinea | 1961 | 0.147 | 0.35 | 0.297 | 0.717 | 1.166 | 0.126 | 1.509 | 0.585 |
|  | 2022 | 0.443 | 0.54 | 0.368 | 1.27 | 1.676 | 0.811 | 0.422 | 0.629 |
| Guinea-Bissau | 1961 | 0.403 | 0.144 | 0.26 | 0.93 | 0.92 | 0.057 | 0.1 | 0.459 |
|  | 2022 | 0.415 | 0.196 | 0.266 | 1.277 | 1.359 | 0.112 | 0.131 | 0.501 |
| Guyana | 1961 | 0.837 | 0.537 | 0.207 | 0.677 | 1.078 | 1.696 | 0.091 | 0.558 |
|  | 2022 | 1.953 | 1.31 | 0.278 | 0.564 | 1.231 | 1.453 | 1.765 | 0.807 |
| Haiti | 1961 | 0.273 | 0.675 | 0.47 | 0.19 | 0.978 | 1.1 | 0.218 | 0.467 |
|  | 2022 | 0.415 | 0.815 | 0.276 | 0.579 | 0.983 | 0.963 | 0.125 | 0.532 |
| Honduras | 1961 | 0.56 | 0.713 | 0.377 | 0.25 | 0.974 | 1.078 | 0.082 | 0.493 |
|  | 2022 | 1.018 | 0.284 | 0.387 | 1.732 | 0.985 | 1.782 | 0.226 | 0.647 |
| Hungary | 1961 | 1.827 | 0.519 | 0.1 | 1.293 | 1.155 | 1.278 | 0.527 | 0.691 |
|  | 2022 | 2.197 | 0.626 | 0.091 | 3.157 | 0.814 | 1.918 | 0.614 | 0.691 |
| Iceland | 1961 | 4.473 | 0.394 | 0.05 | 1.393 | 0.674 | 2.435 | 0.073 | 0.532 |
|  | 2022 | 4.02 | 0.852 | 0.179 | 1.633 | 0.918 | 1.51 | 0.594 | 0.757 |
| India | 1961 | 0.3 | 0.3 | 0.767 | 0.417 | 1.11 | 0.835 | 0.218 | 0.5 |
|  | 2022 | 0.868 | 0.637 | 0.654 | 1.016 | 1.196 | 0.924 | 0.704 | 0.81 |
| Indonesia | 1961 | 0.167 | 0.488 | 0.273 | 0.263 | 1.206 | 0.504 | 0.164 | 0.392 |
|  | 2022 | 0.863 | 0.928 | 0.522 | 1.366 | 1.276 | 1.333 | 0.419 | 0.789 |
| Iran | 1961 | 0.603 | 0.55 | 0.223 | 0.227 | 0.927 | 0.987 | 0.264 | 0.466 |
|  | 2022 | 0.878 | 1.222 | 0.401 | 1.282 | 1.309 | 1.341 | 0.661 | 0.823 |
| Iraq | 1961 | 0.683 | 0.375 | 0.127 | 0.493 | 0.859 | 0.957 | 0.564 | 0.517 |
|  | 2022 | 0.463 | 0.748 | 0.507 | 1.214 | 1.348 | 0.957 | 0.627 | 0.724 |
| Ireland | 1961 | 2.847 | 0.369 | 0.073 | 1.45 | 1.141 | 2.261 | 0.245 | 0.615 |
|  | 2022 | 4.324 | 0.674 | 0.204 | 2.004 | 0.878 | 1.978 | 0.729 | 0.747 |
| Israel | 1961 | 1.463 | 1.087 | 0.327 | 1.157 | 1.084 | 1.496 | 0.855 | 0.864 |
|  | 2022 | 3.034 | 1.315 | 0.76 | 2.201 | 1.047 | 1.617 | 1.249 | 0.96 |
| Italy | 1961 | 1.307 | 0.838 | 0.257 | 1.243 | 1.228 | 1.061 | 0.718 | 0.802 |
|  | 2022 | 2.807 | 0.991 | 0.42 | 2.846 | 0.968 | 1.598 | 0.511 | 0.815 |
| Jamaica | 1961 | 0.763 | 1.225 | 0.083 | 0.62 | 0.728 | 1.674 | 0.109 | 0.551 |
|  | 2022 | 1.69 | 0.782 | 0.147 | 1.277 | 1.038 | 1.8 | 0.744 | 0.779 |
| Japan | 1961 | 0.743 | 0.2 | 0.54 | 0.38 | 1.433 | 0.761 | 0.564 | 0.571 |
|  | 2022 | 1.82 | 0.342 | 0.505 | 1.369 | 0.903 | 1.208 | 0.707 | 0.743 |
| Jordan | 1961 | 0.363 | 0.869 | 0.263 | 0.653 | 1.006 | 1.143 | 1.136 | 0.691 |
|  | 2022 | 1.041 | 0.395 | 0.404 | 1.572 | 0.937 | 1.628 | 0.7 | 0.739 |
| Kazakhstan | 1992 | 2.183 | 0.112 | 0.02 | 0.827 | 1.472 | 0.943 | 0.3 | 0.543 |
|  | 2022 | 3.128 | 0.766 | 0.532 | 1.844 | 0.896 | 1.357 | 1.523 | 0.866 |
| Kenya | 1961 | 0.783 | 0.637 | 0.817 | 0.14 | 1.212 | 0.609 | 0.164 | 0.59 |
|  | 2022 | 0.745 | 0.67 | 0.589 | 0.699 | 0.917 | 1.091 | 0.391 | 0.669 |
| Kiribati | 1961 | 0.783 | 5.281 | 0.083 | 0.653 | 0.747 | 0.922 | 0.345 | 0.602 |
|  | 2022 | 1.348 | 5.096 | 0.111 | 0.975 | 0.888 | 1.912 | 0.285 | 0.71 |
| Kuwait | 1961 | 1.57 | 0.644 | 0.14 | 1.093 | 0.924 | 1.565 | 0.836 | 0.757 |
|  | 2022 | 2.082 | 1.58 | 0.344 | 1.62 | 1.232 | 1.475 | 0.987 | 0.889 |
| Kyrgyzstan | 1992 | 1.9 | 0.219 | 0.047 | 0.703 | 1.266 | 1.113 | 0.536 | 0.584 |
|  | 2022 | 2.162 | 0.328 | 0.647 | 0.592 | 1.086 | 0.876 | 1.39 | 0.761 |
| Lao People's Democratic Republic | 1961 | 0.26 | 0.138 | 0.117 | 0.093 | 1.474 | 0.035 | 0.127 | 0.289 |
|  | 2022 | 0.831 | 1.162 | 0.197 | 0.346 | 1.831 | 0.911 | 1.263 | 0.729 |
| Latvia | 1992 | 2.677 | 0.212 | 0 | 1.197 | 1.323 | 1.678 | 0.5 | 0.785 |
|  | 2022 | 2.808 | 0.545 | 0.143 | 2.326 | 0.783 | 1.645 | 0.596 | 0.678 |
| Lebanon | 1961 | 0.837 | 1.138 | 0.46 | 0.883 | 1.082 | 0.909 | 0.627 | 0.801 |
|  | 2022 | 1.232 | 0.783 | 0.547 | 1.4 | 1.016 | 2.05 | 0.91 | 0.873 |
| Lesotho | 1961 | 0.44 | 0.112 | 0.357 | 0.073 | 1.364 | 0.478 | 0.082 | 0.344 |
|  | 2022 | 0.77 | 0.142 | 0.132 | 0.353 | 1.014 | 0.573 | 0.151 | 0.425 |
| Liberia | 1961 | 0.307 | 0.556 | 0.17 | 0.467 | 1.358 | 0.1 | 0.273 | 0.462 |
|  | 2022 | 0.446 | 0.325 | 0.127 | 0.738 | 1.298 | 0.337 | 0.19 | 0.471 |
| Libya | 2010 | 1.208 | 1.046 | 0.219 | 1.724 | 1.206 | 1.541 | 1.089 | 0.87 |
|  | 2022 | 1.555 | 1.177 | 0.48 | 1.708 | 1.073 | 1.525 | 1.183 | 0.913 |
| Lithuania | 1992 | 2.147 | 0.231 | 0.053 | 1.117 | 1.349 | 1.157 | 0.418 | 0.617 |
|  | 2022 | 3.405 | 0.542 | 0.297 | 1.403 | 0.93 | 1.679 | 0.664 | 0.739 |
| Luxembourg | 2000 | 3.58 | 1.044 | 0.043 | 1.407 | 0.727 | 1.257 | 0.555 | 0.721 |
|  | 2022 | 3.271 | 0.784 | 0.153 | 1.254 | 0.868 | 1.266 | 0.707 | 0.752 |
| Madagascar | 1961 | 0.897 | 0.488 | 0.253 | 0.173 | 1.547 | 0.396 | 0.191 | 0.5 |
|  | 2022 | 0.228 | 0.356 | 0.062 | 0.264 | 1.375 | 0.606 | 0.092 | 0.334 |
| Malawi | 1961 | 0.137 | 0.331 | 1.19 | 0.14 | 1.252 | 0.113 | 0.164 | 0.462 |
|  | 2022 | 0.715 | 1.181 | 0.521 | 0.39 | 1.53 | 0.477 | 0.511 | 0.689 |
| Malaysia | 1961 | 0.74 | 0.944 | 0.19 | 0.7 | 1.235 | 1.3 | 0.136 | 0.618 |
|  | 2022 | 1.77 | 0.592 | 0.29 | 1.851 | 1.038 | 1.747 | 0.54 | 0.737 |
| Maldives | 1961 | 0.13 | 0.688 | 0.133 | 0.513 | 0.689 | 1.522 | 0.409 | 0.427 |
|  | 2022 | 1.947 | 0.773 | 0.51 | 0.533 | 0.952 | 1.041 | 0.888 | 0.776 |
| Mali | 1961 | 0.623 | 0.119 | 0.283 | 0.273 | 1.051 | 0.113 | 0.155 | 0.409 |
|  | 2022 | 0.442 | 0.698 | 0.431 | 0.546 | 1.75 | 0.384 | 0.771 | 0.648 |
| Malta | 1961 | 1.5 | 0.388 | 0.463 | 1.233 | 1.154 | 1.591 | 0.464 | 0.719 |
|  | 2022 | 2.302 | 0.691 | 0.205 | 1.567 | 1.019 | 1.976 | 0.828 | 0.787 |
| Marshall Islands | 2019 | 1.694 | 3.652 | 0.064 | 1.082 | 0.987 | 0.711 | 0.742 | 0.799 |
|  | 2022 | 2.396 | 3.659 | 0.086 | 1.504 | 0.881 | 0.901 | 0.685 | 0.775 |
| Mauritania | 1961 | 2.167 | 0.425 | 0.487 | 0.28 | 0.946 | 0.857 | 0.018 | 0.526 |
|  | 2022 | 1.372 | 0.31 | 0.302 | 0.874 | 1.458 | 1.867 | 0.395 | 0.647 |
| Mauritius | 1961 | 0.523 | 0.094 | 0.307 | 1.05 | 1.133 | 1.809 | 0.164 | 0.515 |
|  | 2022 | 1.69 | 0.397 | 0.405 | 1.879 | 1.124 | 1.493 | 0.589 | 0.732 |
| Mexico | 1961 | 0.843 | 0.438 | 0.623 | 0.547 | 1.147 | 1.061 | 0.118 | 0.595 |
|  | 2022 | 2.27 | 1.037 | 0.379 | 1.451 | 1.177 | 1.746 | 0.432 | 0.802 |
| Micronesia (Federated States of) | 2019 | 1.457 | 2.888 | 0.028 | 1.239 | 0.95 | 1.045 | 0.214 | 0.699 |
|  | 2022 | 1.43 | 3.655 | 0.05 | 1.16 | 0.891 | 1.072 | 0.199 | 0.69 |
| Mongolia | 1961 | 3.107 | 0.019 | 0.023 | 0.437 | 0.774 | 0.126 | 0.036 | 0.382 |
|  | 2022 | 3.98 | 0.247 | 0.073 | 1.21 | 0.872 | 0.733 | 0.494 | 0.615 |
| Montenegro | 2006 | 3.1 | 0.981 | 0.197 | 1.047 | 1.037 | 1.509 | 1.018 | 0.863 |
|  | 2022 | 3.805 | 0.789 | 0.306 | 0.986 | 1.039 | 1.287 | 0.782 | 0.81 |
| Morocco | 1961 | 0.393 | 0.294 | 0.113 | 0.52 | 1.184 | 1.187 | 0.209 | 0.422 |
|  | 2022 | 0.937 | 1.052 | 0.386 | 1.199 | 1.69 | 1.426 | 0.633 | 0.826 |
| Mozambique | 1961 | 0.17 | 0.256 | 0.293 | 0.237 | 1.283 | 0.283 | 0.091 | 0.341 |
|  | 2022 | 0.288 | 0.228 | 0.243 | 0.769 | 1.349 | 0.402 | 0.408 | 0.489 |
| Myanmar | 1961 | 0.28 | 0.288 | 0.197 | 0.45 | 0.909 | 0.2 | 0.127 | 0.375 |
|  | 2022 | 0.712 | 0.486 | 0.779 | 0.565 | 1.583 | 0.752 | 0.66 | 0.7 |
| Namibia | 1961 | 0.953 | 0.225 | 0.41 | 0.733 | 1.077 | 1.196 | 0.136 | 0.576 |
|  | 2022 | 0.925 | 0.354 | 0.28 | 0.921 | 1.176 | 1.405 | 0.185 | 0.611 |
| Nauru | 2019 | 2.653 | 2.435 | 0.412 | 1.071 | 0.567 | 2.11 | 0.215 | 0.699 |
|  | 2022 | 2.514 | 2.381 | 0.388 | 1.133 | 0.589 | 2.142 | 0.303 | 0.713 |
| Nepal | 1961 | 0.45 | 0.062 | 0.207 | 0.263 | 1.246 | 0.048 | 0.045 | 0.338 |
|  | 2022 | 0.66 | 0.474 | 0.439 | 1.193 | 1.604 | 0.46 | 0.993 | 0.761 |
| Netherlands | 1961 | 2.363 | 0.544 | 0.107 | 2.18 | 0.842 | 2 | 0.473 | 0.661 |
|  | 2009 | 3.343 | 0.825 | 0.223 | 1.547 | 0.719 | 1.961 | 0.791 | 0.76 |
| Netherlands (Kingdom of the) | 2010 | 3.14 | 0.888 | 0.189 | 1.706 | 0.752 | 2.02 | 0.667 | 0.749 |
|  | 2022 | 3.596 | 1.26 | 0.279 | 2.001 | 0.762 | 1.936 | 0.721 | 0.794 |
| Netherlands Antilles (former) | 1961 | 2.33 | 0.55 | 0.16 | 1.44 | 1.026 | 1.465 | 0.282 | 0.665 |
|  | 2009 | 2.493 | 0.419 | 0.063 | 0.717 | 0.991 | 2.109 | 0.455 | 0.607 |
| New Caledonia | 1961 | 1.96 | 1.644 | 0.053 | 0.85 | 1.065 | 1.352 | 0.282 | 0.698 |
|  | 2022 | 2.288 | 0.887 | 0.226 | 1.322 | 0.918 | 1.211 | 0.45 | 0.747 |
| New Zealand | 1961 | 2.8 | 0.631 | 0.167 | 1.33 | 0.736 | 2.083 | 0.509 | 0.674 |
|  | 2022 | 2.174 | 0.849 | 0.372 | 1.565 | 0.896 | 2.084 | 0.661 | 0.796 |
| Nicaragua | 1961 | 0.773 | 0.275 | 0.587 | 0.263 | 0.85 | 1.478 | 0.073 | 0.47 |
|  | 2022 | 1.316 | 0.109 | 0.518 | 0.818 | 1.215 | 1.598 | 0.222 | 0.611 |
| Niger | 1961 | 0.49 | 0.081 | 0.36 | 0.167 | 1.08 | 0.096 | 0.118 | 0.369 |
|  | 2022 | 0.391 | 0.323 | 1.975 | 0.446 | 1.339 | 0.255 | 0.935 | 0.682 |
| Nigeria | 1961 | 0.153 | 0.281 | 0.517 | 1.037 | 1.108 | 0.07 | 0.355 | 0.551 |
|  | 2022 | 0.232 | 0.735 | 0.721 | 1.024 | 1.402 | 0.359 | 0.451 | 0.69 |
| North Korea | 1961 | 0.403 | 0.069 | 0.707 | 0.09 | 1.204 | 0.057 | 0.5 | 0.461 |
|  | 2018 | 0.376 | 0.486 | 0.418 | 0.495 | 1.162 | 0.182 | 0.828 | 0.6 |
| North Macedonia | 1992 | 1.07 | 0.569 | 0.273 | 0.817 | 0.995 | 0.922 | 1.027 | 0.776 |
|  | 2022 | 1.63 | 0.982 | 0.435 | 1.64 | 0.973 | 1.554 | 1.86 | 0.898 |
| Norway | 1961 | 2.56 | 0.569 | 0.113 | 1.323 | 0.821 | 1.913 | 0.282 | 0.631 |
|  | 2022 | 2.614 | 0.796 | 0.822 | 1.817 | 0.973 | 1.345 | 0.541 | 0.855 |
| Oman | 1990 | 1.29 | 1.538 | 0.123 | 0.9 | 0.917 | 1.004 | 0.673 | 0.769 |
|  | 2022 | 1.984 | 2.924 | 0.391 | 1.681 | 0.69 | 1.24 | 1.48 | 0.847 |
| Pakistan | 1961 | 0.78 | 0.181 | 0.48 | 0.383 | 0.972 | 0.943 | 0.1 | 0.483 |
|  | 2022 | 1.445 | 0.287 | 0.162 | 1.34 | 0.982 | 1.453 | 0.251 | 0.614 |
| Panama | 1961 | 0.903 | 0.675 | 0.22 | 0.533 | 0.993 | 1.183 | 0.155 | 0.58 |
|  | 2022 | 1.756 | 0.837 | 0.268 | 1.67 | 1.16 | 1.11 | 0.306 | 0.735 |
| Papua New Guinea | 2010 | 1.082 | 3.401 | 0.039 | 0.704 | 0.747 | 0.386 | 0.517 | 0.668 |
|  | 2022 | 0.936 | 2.942 | 0.043 | 0.768 | 0.903 | 0.461 | 0.439 | 0.682 |
| Paraguay | 1961 | 1.267 | 1.056 | 0.307 | 0.55 | 0.96 | 0.587 | 0.3 | 0.686 |
|  | 2022 | 1.228 | 0.552 | 0.407 | 1.285 | 1.463 | 0.791 | 0.391 | 0.725 |
| Peru | 1961 | 0.643 | 0.338 | 0.287 | 0.62 | 1.044 | 1.2 | 0.309 | 0.533 |
|  | 2022 | 1.629 | 1.246 | 0.32 | 0.821 | 1.212 | 0.87 | 0.509 | 0.775 |
| Philippines | 1961 | 0.62 | 1.069 | 0.063 | 0.263 | 0.975 | 0.613 | 0.455 | 0.563 |
|  | 2022 | 0.965 | 0.687 | 0.134 | 0.769 | 1.558 | 1.001 | 0.45 | 0.668 |
| Poland | 1961 | 2.027 | 0.15 | 0.057 | 1.263 | 1.546 | 1.378 | 0.482 | 0.615 |
|  | 2022 | 2.897 | 0.596 | 0.176 | 2.209 | 1.033 | 2.122 | 0.706 | 0.746 |
| Portugal | 1961 | 0.94 | 0.744 | 0.38 | 0.96 | 1.028 | 0.796 | 0.555 | 0.763 |
|  | 2022 | 2.923 | 0.991 | 0.235 | 2.139 | 0.935 | 1.096 | 0.706 | 0.811 |
| Qatar | 2019 | 2.076 | 1.011 | 0.509 | 1.547 | 1.305 | 1.141 | 1.148 | 0.918 |
|  | 2022 | 2.136 | 1.09 | 0.57 | 1.698 | 1.24 | 1.219 | 1.192 | 0.928 |
| Republic of Korea | 1961 | 0.16 | 0.038 | 0.24 | 0.057 | 1.595 | 0.078 | 0.536 | 0.338 |
|  | 2022 | 1.906 | 0.444 | 0.461 | 2.244 | 0.999 | 2.066 | 1.719 | 0.817 |
| Republic of Moldova | 1992 | 1.773 | 0.456 | 0.153 | 0.51 | 1.106 | 1.026 | 0.564 | 0.614 |
|  | 2022 | 1.575 | 1.054 | 0.422 | 1.348 | 0.911 | 1.766 | 0.742 | 0.846 |
| Romania | 1961 | 1.177 | 0.319 | 0.237 | 0.69 | 1.648 | 0.461 | 0.464 | 0.618 |
|  | 2022 | 2.705 | 0.852 | 0.197 | 1.975 | 1.175 | 1.344 | 0.687 | 0.789 |
| Russian Federation | 1992 | 1.897 | 0.262 | 0.1 | 1.137 | 1.228 | 1.417 | 0.5 | 0.644 |
|  | 2022 | 2.556 | 0.628 | 0.174 | 1.751 | 1.18 | 1.745 | 0.636 | 0.74 |
| Rwanda | 1961 | 0.103 | 3.394 | 1.093 | 0.037 | 0.697 | 0 | 0.155 | 0.499 |
|  | 2022 | 0.248 | 1.388 | 0.989 | 0.413 | 1.197 | 0.59 | 0.284 | 0.656 |
| Saint Kitts and Nevis | 1961 | 0.627 | 0.631 | 0.093 | 0.83 | 0.608 | 1.665 | 0.073 | 0.477 |
|  | 2022 | 2.044 | 1.198 | 0.344 | 1.636 | 0.7 | 1.977 | 0.249 | 0.715 |
| Saint Lucia | 1961 | 0.733 | 1.794 | 0.047 | 0.543 | 0.54 | 1.213 | 0.064 | 0.488 |
|  | 2022 | 2.247 | 1.023 | 0.29 | 0.788 | 0.793 | 1.696 | 0.268 | 0.69 |
| Saint Vincent and the Grenadines | 1961 | 0.587 | 1.144 | 0.083 | 0.497 | 0.775 | 1.339 | 0.045 | 0.498 |
|  | 2022 | 2.48 | 1.192 | 0.441 | 1.232 | 0.798 | 1.238 | 0.403 | 0.774 |
| Samoa | 1961 | 0.957 | 4.056 | 0 | 0.527 | 0.572 | 0.883 | 0.018 | 0.679 |
|  | 2022 | 2.13 | 2.67 | 0.062 | 1.266 | 0.992 | 1.517 | 0.156 | 0.702 |
| Sao Tome and Principe | 1961 | 0.263 | 4.125 | 0.573 | 0.163 | 0.942 | 0.574 | 0.109 | 0.509 |
|  | 2022 | 0.584 | 0.496 | 0.082 | 1.138 | 1.247 | 0.849 | 0.192 | 0.559 |
| Saudi Arabia | 1961 | 0.403 | 1.112 | 0.107 | 0.257 | 1.042 | 0.287 | 0.245 | 0.502 |
|  | 2022 | 1.402 | 1.681 | 0.253 | 1.666 | 1.292 | 1.373 | 0.636 | 0.815 |
| Senegal | 1961 | 0.54 | 0.119 | 0.51 | 0.673 | 1.312 | 0.843 | 0.145 | 0.498 |
|  | 2022 | 0.595 | 0.178 | 0.685 | 1.288 | 1.497 | 0.772 | 0.922 | 0.73 |
| Serbia | 2006 | 1.893 | 0.844 | 0.397 | 1.057 | 0.903 | 1.296 | 0.782 | 0.821 |
|  | 2022 | 2.595 | 1.42 | 0.453 | 1.108 | 1.493 | 0.517 | 0.648 | 0.85 |
| Serbia and Montenegro | 1992 | 2.387 | 0.662 | 0.317 | 1.993 | 0.916 | 0.861 | 0.636 | 0.755 |
|  | 2005 | 2.48 | 0.588 | 0.347 | 1.79 | 0.613 | 1.283 | 0.755 | 0.717 |
| Seychelles | 2010 | 1.338 | 0.743 | 0.222 | 0.57 | 1.298 | 1.595 | 0.577 | 0.685 |
|  | 2022 | 2.105 | 1.639 | 0.361 | 0.763 | 1.093 | 1.152 | 0.915 | 0.84 |
| Sierra Leone | 1961 | 0.213 | 0.181 | 0.69 | 1.32 | 0.791 | 0.357 | 0.382 | 0.543 |
|  | 2022 | 0.396 | 0.231 | 0.436 | 0.828 | 1.431 | 0.262 | 0.37 | 0.543 |
| Slovakia | 1993 | 1.927 | 0.338 | 0.193 | 1.6 | 0.897 | 1.383 | 0.455 | 0.647 |
|  | 2022 | 1.851 | 0.511 | 0.173 | 3.108 | 0.729 | 1.138 | 0.49 | 0.65 |
| Slovenia | 1992 | 1.7 | 0.394 | 0.12 | 1.543 | 0.947 | 0.635 | 0.327 | 0.631 |
|  | 2022 | 2.085 | 0.998 | 0.234 | 1.479 | 1.015 | 1.238 | 0.641 | 0.812 |
| Solomon Islands | 1961 | 0.537 | 1.725 | 0.257 | 0.14 | 1.347 | 0.274 | 0.136 | 0.512 |
|  | 2022 | 0.573 | 1.146 | 0.273 | 0.661 | 1.243 | 0.698 | 0.072 | 0.596 |
| Somalia | 2010 | 1.268 | 0.157 | 0.144 | 0.372 | 0.621 | 1.006 | 0.051 | 0.391 |
|  | 2022 | 0.879 | 0.161 | 0.162 | 0.47 | 0.665 | 2.434 | 0.086 | 0.404 |
| South Africa | 1961 | 1.197 | 0.181 | 0.143 | 0.627 | 1.335 | 1.665 | 0.336 | 0.548 |
|  | 2022 | 1.501 | 0.279 | 0.119 | 1.553 | 1.09 | 1.27 | 0.295 | 0.616 |
| South Sudan | 2019 | 1.692 | 0.324 | 1.279 | 0.569 | 0.937 | 0.39 | 0.322 | 0.692 |
|  | 2022 | 1.727 | 0.308 | 1.244 | 0.588 | 0.993 | 0.393 | 0.322 | 0.702 |
| Spain | 1961 | 1.087 | 0.456 | 0.447 | 1.253 | 1.118 | 0.87 | 0.955 | 0.81 |
|  | 2022 | 2.881 | 0.679 | 0.424 | 2.519 | 0.797 | 1.438 | 0.744 | 0.774 |
| Sri Lanka | 1961 | 0.29 | 1.781 | 0.237 | 0.297 | 1.107 | 0.809 | 0.191 | 0.502 |
|  | 2022 | 0.677 | 0.918 | 0.297 | 0.344 | 1.552 | 1.461 | 0.487 | 0.62 |
| Sudan | 2012 | 1.177 | 0.841 | 0.513 | 0.676 | 1.159 | 1.468 | 0.514 | 0.757 |
|  | 2022 | 0.978 | 0.725 | 0.803 | 0.953 | 1.125 | 1.481 | 0.525 | 0.831 |
| Sudan (former) | 1961 | 0.927 | 0.362 | 0.38 | 0.457 | 0.724 | 0.522 | 0.2 | 0.508 |
|  | 2009 | 1.703 | 0.575 | 0.44 | 0.593 | 0.953 | 1.057 | 0.455 | 0.669 |
| Suriname | 1961 | 0.773 | 0.188 | 0.187 | 0.733 | 0.844 | 1.283 | 0.118 | 0.474 |
|  | 2022 | 1.226 | 0.488 | 0.16 | 1.044 | 1.005 | 2.12 | 0.331 | 0.663 |
| Sweden | 1961 | 2.483 | 0.531 | 0.08 | 2.11 | 0.657 | 1.961 | 0.209 | 0.58 |
|  | 2022 | 2.845 | 0.589 | 0.277 | 2.177 | 0.865 | 1.473 | 0.665 | 0.733 |
| Switzerland | 1961 | 2.89 | 0.981 | 0.227 | 1.797 | 0.986 | 2.261 | 0.464 | 0.776 |
|  | 2022 | 2.99 | 0.81 | 0.348 | 2.384 | 0.816 | 1.61 | 0.688 | 0.777 |
| Syrian Arab Republic | 2010 | 0.957 | 0.615 | 0.564 | 1.937 | 1.318 | 1.514 | 0.525 | 0.777 |
|  | 2022 | 0.899 | 0.681 | 0.435 | 1.216 | 1.104 | 0.582 | 0.545 | 0.76 |
| Tajikistan | 1992 | 0.833 | 0.231 | 0.037 | 0.81 | 1.122 | 0.474 | 0.627 | 0.59 |
|  | 2022 | 1.421 | 0.427 | 0.221 | 1.251 | 1.215 | 0.817 | 1.66 | 0.775 |
| Thailand | 1961 | 0.523 | 1.231 | 0.083 | 0.117 | 1.236 | 0.23 | 0.282 | 0.501 |
|  | 2022 | 0.995 | 0.618 | 0.207 | 0.928 | 1.24 | 2.11 | 0.315 | 0.677 |
| Timor-Leste | 1961 | 1.173 | 0.506 | 0.23 | 0.137 | 1.008 | 0.03 | 0.173 | 0.508 |
|  | 2022 | 0.775 | 0.167 | 0.333 | 0.944 | 1.188 | 0.707 | 0.232 | 0.575 |
| Togo | 1961 | 0.18 | 0.125 | 0.36 | 0.47 | 1.415 | 0.096 | 0.136 | 0.379 |
|  | 2022 | 0.29 | 0.064 | 0.479 | 1.029 | 1.573 | 0.61 | 0.206 | 0.507 |
| Tonga | 2019 | 3.026 | 1.136 | 0.294 | 0.895 | 0.94 | 0.804 | 1.388 | 0.855 |
|  | 2022 | 2.893 | 1.159 | 0.301 | 1.062 | 0.869 | 0.858 | 1.501 | 0.862 |
| Trinidad and Tobago | 1961 | 1.023 | 0.406 | 0.293 | 0.853 | 0.978 | 1.57 | 0.118 | 0.608 |
|  | 2022 | 1.537 | 0.576 | 0.385 | 1.683 | 1.02 | 1.76 | 0.355 | 0.719 |
| Tunisia | 1961 | 0.513 | 0.444 | 0.127 | 0.773 | 1.224 | 1.017 | 0.436 | 0.549 |
|  | 2022 | 1.295 | 1.234 | 0.499 | 1.809 | 1.386 | 1.495 | 1.483 | 0.916 |
| Turkey | 1961 | 1.327 | 1.312 | 0.463 | 0.897 | 1.486 | 0.296 | 0.873 | 0.872 |
|  | 2022 | 2.257 | 1.317 | 0.777 | 2.09 | 1.299 | 1.27 | 1.444 | 0.963 |
| Turkmenistan | 1992 | 1.423 | 0.281 | 0.007 | 0.987 | 1.301 | 0.957 | 0.482 | 0.626 |
|  | 2022 | 2.115 | 0.591 | 0.151 | 0.743 | 1.302 | 0.808 | 0.849 | 0.722 |
| Tuvalu | 2019 | 1.729 | 3.433 | 0.041 | 0.7 | 0.809 | 2.198 | 0.431 | 0.664 |
|  | 2022 | 1.626 | 3 | 0.041 | 0.687 | 0.947 | 2.13 | 0.433 | 0.685 |
| Uganda | 1961 | 0.46 | 0.281 | 1.227 | 0.19 | 1.175 | 0.474 | 0.118 | 0.508 |
|  | 2022 | 0.496 | 0.011 | 0.884 | 0.757 | 1.118 | 0.558 | 0.205 | 0.559 |
| Ukraine | 1992 | 2.02 | 0.319 | 0.163 | 1.31 | 1.397 | 2.165 | 0.509 | 0.665 |
|  | 2022 | 1.936 | 0.442 | 0.118 | 1.107 | 1.096 | 1.438 | 1.067 | 0.76 |
| United Arab Emirates | 1961 | 2.343 | 0.419 | 0.013 | 1.463 | 1.142 | 1.504 | 0.036 | 0.578 |
|  | 2022 | 1.937 | 0.879 | 0.782 | 2.327 | 1.091 | 1.02 | 0.756 | 0.903 |
| United Kingdom | 1961 | 2.96 | 0.488 | 0.15 | 1.933 | 0.814 | 2.191 | 0.355 | 0.634 |
|  | 2022 | 2.727 | 0.784 | 0.222 | 2.027 | 0.967 | 1.229 | 0.692 | 0.777 |
| United Republic of Tanzania | 1961 | 0.407 | 0.231 | 0.37 | 0.227 | 1.065 | 0.296 | 0.3 | 0.422 |
|  | 2022 | 0.577 | 0.626 | 0.868 | 0.818 | 1.19 | 0.496 | 0.234 | 0.687 |
| United States of America | 1961 | 2.7 | 0.531 | 0.283 | 1.59 | 0.62 | 2.243 | 0.582 | 0.669 |
|  | 2022 | 3.263 | 0.964 | 0.456 | 2.891 | 0.793 | 2.625 | 0.662 | 0.813 |
| Uruguay | 1961 | 3.463 | 0.394 | 0.09 | 0.873 | 0.763 | 1.63 | 0.236 | 0.559 |
|  | 2022 | 2.727 | 0.7 | 0.157 | 1.557 | 1.018 | 1.918 | 0.489 | 0.724 |
| USSR | 1961 | 1.73 | 0.169 | 0.15 | 0.823 | 1.528 | 1.348 | 0.382 | 0.587 |
|  | 1991 | 2.197 | 0.306 | 0.117 | 1.113 | 1.23 | 1.53 | 0.473 | 0.649 |
| Uzbekistan | 1992 | 1.407 | 0.281 | 0.023 | 1.083 | 1.447 | 0.543 | 0.864 | 0.695 |
|  | 2022 | 2.597 | 1.078 | 0.127 | 1.139 | 1.239 | 1.07 | 1.832 | 0.854 |
| Vanuatu | 1961 | 1.407 | 2.306 | 0.073 | 0.563 | 1.091 | 0.548 | 0.255 | 0.649 |
|  | 2022 | 0.979 | 3.279 | 0.348 | 0.975 | 1.155 | 0.62 | 0.313 | 0.769 |
| Venezuela | 1961 | 1.003 | 1.181 | 0.297 | 0.823 | 0.768 | 1.526 | 0.109 | 0.666 |
|  | 2022 | 1.106 | 0.594 | 0.351 | 1.354 | 0.856 | 1.32 | 0.314 | 0.686 |
| Viet Nam | 1961 | 0.417 | 0.306 | 0.113 | 0.09 | 1.363 | 0.191 | 0.273 | 0.366 |
|  | 2022 | 1.472 | 0.766 | 0.609 | 0.539 | 1.435 | 1.168 | 1.213 | 0.819 |
| Yemen | 1961 | 0.36 | 0.325 | 0.223 | 0.273 | 1.165 | 0.457 | 0.091 | 0.379 |
|  | 2022 | 0.411 | 0.393 | 0.225 | 0.561 | 1.135 | 1.077 | 0.181 | 0.462 |
| Yugoslav SFR | 1961 | 1.117 | 0.588 | 0.317 | 0.9 | 1.656 | 0.835 | 0.345 | 0.692 |
|  | 1991 | 1.793 | 0.494 | 0.207 | 1.78 | 1.468 | 1.474 | 0.518 | 0.703 |
| Zambia | 1961 | 0.39 | 0.088 | 0.41 | 0.127 | 1.435 | 0.265 | 0.191 | 0.368 |
|  | 2022 | 0.462 | 0.039 | 0.464 | 0.701 | 1.277 | 0.531 | 0.149 | 0.469 |
| Zimbabwe | 1961 | 0.637 | 0.112 | 0.407 | 0.183 | 1.344 | 0.426 | 0.109 | 0.408 |
|  | 2022 | 1.238 | 0.158 | 0.134 | 1.184 | 0.868 | 0.767 | 0.088 | 0.541 |

| Table S3. Regional HDBI and food supply as percentage of reference intake (1961 and 2022). | | | | | | | | | |
| --- | --- | --- | --- | --- | --- | --- | --- | --- | --- |
| Region | Year | ASF | Fruits | LNS | Oils & Fats | Starchy Staples | Sugars | Vegetables | HDBI |
| East Asia & Pacific | 1961 | 0.262 | 0.171 | 0.422 | 0.161 | 1.067 | 0.229 | 0.492 | 0.418 |
|  | 2022 | 1.727 | 0.832 | 0.524 | 0.945 | 1.419 | 0.725 | 2.057 | 0.883 |
| Europe & Central Asia | 1961 | 1.877 | 0.438 | 0.173 | 1.297 | 1.238 | 1.374 | 0.482 | 0.682 |
|  | 2022 | 2.720 | 0.770 | 0.272 | 2.122 | 0.973 | 1.584 | 0.794 | 0.802 |
| Latin America & Caribbean | 1961 | 1.063 | 0.547 | 0.488 | 0.547 | 0.957 | 1.538 | 0.222 | 0.627 |
|  | 2022 | 2.147 | 0.915 | 0.357 | 1.668 | 1.009 | 1.685 | 0.402 | 0.779 |
| North America | 1961 | 2.677 | 0.537 | 0.270 | 1.597 | 0.622 | 2.226 | 0.573 | 0.667 |
|  | 2022 | 3.168 | 0.954 | 0.478 | 2.904 | 0.799 | 2.550 | 0.671 | 0.817 |
| South Asia | 1961 | 0.340 | 0.306 | 0.647 | 0.380 | 1.151 | 0.787 | 0.209 | 0.480 |
|  | 2022 | 0.911 | 0.585 | 0.534 | 1.026 | 1.226 | 0.942 | 0.621 | 0.775 |
| Sub-Saharan Africa | 1961 | 0.441 | 0.285 | 0.503 | 0.502 | 1.184 | 0.354 | 0.242 | 0.495 |
|  | 2022 | 0.499 | 0.422 | 0.625 | 0.784 | 1.367 | 0.535 | 0.313 | 0.607 |
| Western Asia & North Africa | 1961 | 0.714 | 0.661 | 0.278 | 0.608 | 1.164 | 0.650 | 0.525 | 0.631 |
|  | 2022 | 1.271 | 1.051 | 0.429 | 1.343 | 1.371 | 1.240 | 0.903 | 0.889 |
| World* | 1961 | 0.893 | 0.338 | 0.410 | 0.620 | 1.091 | 0.848 | 0.391 | 0.609 |
|  | 2022 | 1.519 | 0.734 | 0.492 | 1.248 | 1.246 | 1.046 | 1.020 | 0.871 |
| Average of all countries** | 1961 | 1.031 | 0.632 | 0.296 | 0.697 | 1.068 | 0.974 | 0.299 | 0.548 |
|  | 2022 | 1.691 | 0.856 | 0.390 | 1.331 | 1.103 | 1.249 | 0.634 | 0.717 |
| * Denotes average values calculated from pooled global food supply.  ** Denotes average values calculated from national data. | | | | | | | | | |

| Table S4. IMPACT projections for regional HDBI and food supply as percentage of reference intake, increased investments and reference scenario (2010-2050). | | | | | | | | | | |
| --- | --- | --- | --- | --- | --- | --- | --- | --- | --- | --- |
| Region | Scenario | Year | ASF | Fruits | LNS | Oils & Fats | Starchy Staples | Sugars | Veg. | HDBI |
| East Asia & Pacific | Incr. Investments | 2010 | 1.241 | 0.968 | 0.343 | 1.062 | 1.145 | 1.211 | 0.52 | 0.67 |
|  |  | 2020 | 1.393 | 1.043 | 0.362 | 1.138 | 1.168 | 1.319 | 0.58 | 0.7 |
|  |  | 2030 | 1.538 | 1.132 | 0.385 | 1.201 | 1.225 | 1.401 | 0.61 | 0.73 |
|  |  | 2040 | 1.614 | 1.178 | 0.389 | 1.22 | 1.226 | 1.446 | 0.62 | 0.73 |
|  |  | 2050 | 1.665 | 1.211 | 0.391 | 1.231 | 1.211 | 1.478 | 0.63 | 0.74 |
|  | Reference Scenario | 2010 | 1.241 | 0.968 | 0.343 | 1.062 | 1.145 | 1.211 | 0.52 | 0.67 |
|  |  | 2020 | 1.388 | 1.036 | 0.36 | 1.135 | 1.154 | 1.315 | 0.58 | 0.7 |
|  |  | 2030 | 1.499 | 1.092 | 0.37 | 1.184 | 1.155 | 1.391 | 0.61 | 0.72 |
|  |  | 2040 | 1.564 | 1.13 | 0.371 | 1.201 | 1.144 | 1.434 | 0.62 | 0.73 |
|  |  | 2050 | 1.613 | 1.162 | 0.373 | 1.211 | 1.128 | 1.465 | 0.63 | 0.73 |
| Europe & Central Asia | Incr. Investments | 2010 | 2.467 | 0.69 | 0.216 | 1.797 | 0.96 | 1.495 | 0.83 | 0.76 |
|  |  | 2020 | 2.492 | 0.721 | 0.221 | 1.801 | 0.968 | 1.548 | 0.87 | 0.77 |
|  |  | 2030 | 2.539 | 0.755 | 0.231 | 1.829 | 1.011 | 1.592 | 0.9 | 0.78 |
|  |  | 2040 | 2.564 | 0.779 | 0.234 | 1.847 | 1.019 | 1.625 | 0.9 | 0.78 |
|  |  | 2050 | 2.584 | 0.798 | 0.237 | 1.859 | 1.016 | 1.645 | 0.9 | 0.79 |
|  | Reference Scenario | 2010 | 2.467 | 0.69 | 0.216 | 1.797 | 0.96 | 1.495 | 0.83 | 0.76 |
|  |  | 2020 | 2.488 | 0.72 | 0.22 | 1.799 | 0.959 | 1.546 | 0.87 | 0.77 |
|  |  | 2030 | 2.511 | 0.751 | 0.225 | 1.821 | 0.962 | 1.589 | 0.9 | 0.77 |
|  |  | 2040 | 2.53 | 0.775 | 0.227 | 1.839 | 0.961 | 1.623 | 0.91 | 0.78 |
|  |  | 2050 | 2.548 | 0.793 | 0.229 | 1.85 | 0.955 | 1.643 | 0.91 | 0.78 |
| Latin America & Caribbean | Incr. Investments | 2010 | 1.496 | 0.742 | 0.363 | 1.184 | 1.023 | 1.839 | 0.37 | 0.71 |
|  |  | 2020 | 1.554 | 0.788 | 0.379 | 1.221 | 1.031 | 1.926 | 0.41 | 0.72 |
|  |  | 2030 | 1.627 | 0.828 | 0.404 | 1.26 | 1.065 | 1.981 | 0.43 | 0.74 |
|  |  | 2040 | 1.662 | 0.852 | 0.418 | 1.284 | 1.064 | 2.02 | 0.44 | 0.75 |
|  |  | 2050 | 1.692 | 0.874 | 0.429 | 1.31 | 1.054 | 2.062 | 0.45 | 0.76 |
|  | Reference Scenario | 2010 | 1.496 | 0.742 | 0.363 | 1.184 | 1.023 | 1.839 | 0.37 | 0.71 |
|  |  | 2020 | 1.547 | 0.786 | 0.377 | 1.219 | 1.022 | 1.921 | 0.41 | 0.72 |
|  |  | 2030 | 1.585 | 0.819 | 0.391 | 1.251 | 1.016 | 1.975 | 0.43 | 0.73 |
|  |  | 2040 | 1.61 | 0.841 | 0.4 | 1.273 | 1.006 | 2.013 | 0.44 | 0.74 |
|  |  | 2050 | 1.638 | 0.862 | 0.411 | 1.298 | 0.994 | 2.054 | 0.45 | 0.75 |
| North America | Incr. Investments | 2010 | 2.611 | 0.816 | 0.479 | 2.714 | 0.841 | 2.276 | 0.78 | 0.82 |
|  |  | 2020 | 2.616 | 0.828 | 0.485 | 2.612 | 0.841 | 2.313 | 0.82 | 0.83 |
|  |  | 2030 | 2.651 | 0.843 | 0.506 | 2.565 | 0.876 | 2.333 | 0.85 | 0.84 |
|  |  | 2040 | 2.675 | 0.858 | 0.511 | 2.581 | 0.886 | 2.361 | 0.87 | 0.85 |
|  |  | 2050 | 2.7 | 0.869 | 0.51 | 2.601 | 0.889 | 2.382 | 0.88 | 0.86 |
|  | Reference Scenario | 2010 | 2.611 | 0.816 | 0.479 | 2.714 | 0.841 | 2.276 | 0.78 | 0.82 |
|  |  | 2020 | 2.611 | 0.827 | 0.482 | 2.612 | 0.832 | 2.31 | 0.82 | 0.83 |
|  |  | 2030 | 2.617 | 0.838 | 0.484 | 2.568 | 0.828 | 2.337 | 0.86 | 0.83 |
|  |  | 2040 | 2.633 | 0.853 | 0.485 | 2.588 | 0.829 | 2.369 | 0.88 | 0.84 |
|  |  | 2050 | 2.656 | 0.863 | 0.484 | 2.608 | 0.829 | 2.39 | 0.89 | 0.84 |
| South Asia | Incr. Investments | 2010 | 0.601 | 0.426 | 0.273 | 0.754 | 1.369 | 0.771 | 0.38 | 0.56 |
|  |  | 2020 | 0.667 | 0.527 | 0.295 | 0.792 | 1.405 | 0.888 | 0.47 | 0.61 |
|  |  | 2030 | 0.749 | 0.628 | 0.325 | 0.848 | 1.511 | 0.977 | 0.57 | 0.65 |
|  |  | 2040 | 0.788 | 0.684 | 0.338 | 0.877 | 1.521 | 1.012 | 0.64 | 0.67 |
|  |  | 2050 | 0.816 | 0.726 | 0.348 | 0.905 | 1.502 | 1.029 | 0.71 | 0.69 |
|  | Reference Scenario | 2010 | 0.601 | 0.426 | 0.273 | 0.754 | 1.369 | 0.771 | 0.38 | 0.56 |
|  |  | 2020 | 0.661 | 0.523 | 0.292 | 0.788 | 1.383 | 0.883 | 0.47 | 0.6 |
|  |  | 2030 | 0.711 | 0.607 | 0.308 | 0.826 | 1.394 | 0.96 | 0.56 | 0.64 |
|  |  | 2040 | 0.738 | 0.657 | 0.317 | 0.852 | 1.384 | 0.993 | 0.63 | 0.66 |
|  |  | 2050 | 0.761 | 0.694 | 0.326 | 0.878 | 1.361 | 1.008 | 0.69 | 0.67 |
| Sub-Saharan Africa | Incr. Investments | 2010 | 0.623 | 0.363 | 0.559 | 0.769 | 1.308 | 0.625 | 0.25 | 0.56 |
|  |  | 2020 | 0.686 | 0.397 | 0.584 | 0.799 | 1.344 | 0.646 | 0.28 | 0.58 |
|  |  | 2030 | 0.803 | 0.451 | 0.646 | 0.854 | 1.436 | 0.673 | 0.3 | 0.62 |
|  |  | 2040 | 0.887 | 0.485 | 0.668 | 0.881 | 1.43 | 0.695 | 0.32 | 0.64 |
|  |  | 2050 | 0.965 | 0.517 | 0.681 | 0.909 | 1.406 | 0.719 | 0.34 | 0.65 |
|  | Reference Scenario | 2010 | 0.623 | 0.363 | 0.559 | 0.769 | 1.308 | 0.625 | 0.25 | 0.56 |
|  |  | 2020 | 0.679 | 0.393 | 0.577 | 0.794 | 1.322 | 0.643 | 0.28 | 0.58 |
|  |  | 2030 | 0.742 | 0.421 | 0.593 | 0.824 | 1.31 | 0.663 | 0.3 | 0.6 |
|  |  | 2040 | 0.798 | 0.443 | 0.601 | 0.842 | 1.282 | 0.679 | 0.31 | 0.61 |
|  |  | 2050 | 0.859 | 0.468 | 0.611 | 0.862 | 1.255 | 0.699 | 0.32 | 0.62 |
| Western Asia & North Africa | Incr. Investments | 2010 | 1.351 | 0.85 | 0.328 | 1.38 | 1.26 | 1.394 | 0.94 | 0.79 |
|  |  | 2020 | 1.405 | 0.868 | 0.343 | 1.416 | 1.275 | 1.515 | 0.99 | 0.8 |
|  |  | 2030 | 1.494 | 0.896 | 0.36 | 1.476 | 1.339 | 1.643 | 1.03 | 0.81 |
|  |  | 2040 | 1.538 | 0.906 | 0.37 | 1.504 | 1.343 | 1.731 | 1.05 | 0.82 |
|  |  | 2050 | 1.566 | 0.912 | 0.376 | 1.517 | 1.326 | 1.789 | 1.06 | 0.82 |
|  | Reference Scenario | 2010 | 1.351 | 0.85 | 0.328 | 1.38 | 1.26 | 1.394 | 0.94 | 0.79 |
|  |  | 2020 | 1.398 | 0.867 | 0.342 | 1.412 | 1.261 | 1.509 | 0.99 | 0.8 |
|  |  | 2030 | 1.444 | 0.888 | 0.356 | 1.459 | 1.265 | 1.625 | 1.04 | 0.81 |
|  |  | 2040 | 1.475 | 0.897 | 0.365 | 1.484 | 1.256 | 1.707 | 1.06 | 0.81 |
|  |  | 2050 | 1.5 | 0.902 | 0.37 | 1.495 | 1.236 | 1.763 | 1.06 | 0.82 |
| Average of national HDBIs | Incr. Investments | 2010 | 1.425 | 0.653 | 0.372 | 1.237 | 1.144 | 1.236 | 0.55 | 0.68 |
|  |  | 2020 | 1.488 | 0.694 | 0.388 | 1.266 | 1.164 | 1.304 | 0.59 | 0.7 |
|  |  | 2030 | 1.578 | 0.744 | 0.419 | 1.312 | 1.227 | 1.362 | 0.62 | 0.72 |
|  |  | 2040 | 1.63 | 0.773 | 0.43 | 1.335 | 1.228 | 1.401 | 0.64 | 0.73 |
|  |  | 2050 | 1.673 | 0.798 | 0.438 | 1.355 | 1.214 | 1.432 | 0.65 | 0.74 |
|  | Reference Scenario | 2010 | 1.425 | 0.653 | 0.372 | 1.237 | 1.144 | 1.236 | 0.55 | 0.68 |
|  |  | 2020 | 1.482 | 0.691 | 0.385 | 1.263 | 1.15 | 1.3 | 0.59 | 0.7 |
|  |  | 2030 | 1.534 | 0.726 | 0.396 | 1.295 | 1.147 | 1.353 | 0.62 | 0.71 |
|  |  | 2040 | 1.572 | 0.75 | 0.402 | 1.315 | 1.134 | 1.39 | 0.64 | 0.72 |
|  |  | 2050 | 1.608 | 0.772 | 0.409 | 1.331 | 1.118 | 1.419 | 0.65 | 0.72 |

| **Table S5. List of countries and country groups included in IMPACT modeling.** | | | | |
| --- | --- | --- | --- | --- |
|  | IMPACT country  * country or territory is included only as part of an aggregate group  † country group is excluded from Figure 4 | | | |
|  |  | Afghanistan | |  |
|  | 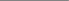 | Angola | |  |
|  | 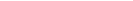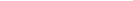 | Albania | |  |
|  |  | Argentina | |  |
|  |  | Armenia | |  |
|  |  | Australia | |  |
|  |  | Austria | |  |
|  |  | Azerbaijan | |  |
|  |  | Burundi | |  |
|  |  | Benin | |  |
|  |  | Burkina Faso | |  |
|  |  | Bangladesh | |  |
|  |  | Bulgaria | |  |
|  |  | Belarus | |  |
|  |  | Baltic States: | |  |
|  |  |  | Estonia* |  |
|  |  |  | Lithuania* |  |
|  |  |  | Latvia* |  |
|  |  | Belgium-Luxembourg | |  |
|  |  | Belize | |  |
|  |  | Bolivia | |  |
|  |  | Brazil | |  |
|  |  | Bhutan | |  |
|  |  | Botswana | |  |
|  |  | Central African Republic | |  |
|  |  | Canada | |  |
|  |  | Chile | |  |
|  |  | China Plus: | |  |
|  |  |  | China* |  |
|  |  |  | Hong Kong* |  |
|  |  |  | Macao* |  |
|  |  |  | Taiwan* |  |
|  |  | Switzerland Plus: | |  |
|  |  |  | Switzerland* |  |
|  |  |  | Liechtenstein* |  |
|  |  | Ivory Coast | |  |
|  |  | Cameroon | |  |
|  |  | Democratic Republic of Congo | | |
|  |  | Congo | |  |
|  |  | Colombia | |  |
|  |  | Other Caribbean†: | |  |
|  |  |  | Aruba* |  |
|  |  |  | Anguilla* |  |
|  |  |  | Netherlands Antilles (obsolete) * | |
|  |  |  | Antigua* |  |
|  |  |  | Bonaire, Sint Eustatius, and Saba* | |
|  |  |  | Bahamas* |  |
|  |  |  | St. Barthelemy* |  |
|  |  |  | Barbados* |  |
|  |  |  | Curacao* |  |
|  |  |  | Cayman Islands* |  |
|  |  |  | Dominica* |  |
|  |  |  | Guadeloupe* |  |
|  |  |  | Grenada* |  |
|  |  |  | St. Kitts and Nevis* |  |
|  |  |  | St. Lucia* |  |
|  |  |  | Saint Martin* |  |
|  |  |  | Montserrat* |  |
|  |  |  | Martinique* |  |
|  |  |  | Puerto Rico* |  |
|  |  |  | Sint Maarten* |  |
|  |  |  | Turks and Caicos Islands* | |
|  |  |  | Trinidad and Tobago* |  |
|  |  |  | St. Vincent and Grenadines* | |
|  |  |  | British Virgin Islands* |  |
|  |  |  | US Virgin Islands* |  |
|  |  | Costa Rica | |  |
|  |  | Cuba | |  |
|  |  | Cyprus | |  |
|  |  | Czech Republic | |  |
|  |  | Germany | |  |
|  |  | Djibouti | |  |
|  |  | Denmark | |  |
|  | 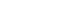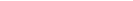 | Dominican Republic | |  |
|  |  | Algeria | |  |
|  |  | Ecuador | |  |
|  |  | Egypt | |  |
|  |  | Eritrea | |  |
|  |  | Ethiopia | | 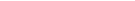 |
|  | 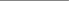 | Fiji | |  |
|  |  | Finland Plus: | |  |
|  |  |  | Aland Islands* |  |
|  | 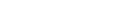 |  | Finland* |  |
|  |  | France Plus: | |  |
|  |  |  | France* |  |
|  |  |  | Monaco* |  |
|  |  | Gabon | |  |
|  |  | Georgia | |  |
|  |  | Ghana | |  |
|  |  | Guinea | |  |
|  |  | Gambia | |  |
|  |  | Guinea-Bissau | |  |
|  |  | Equatorial Guinea | |  |
|  |  | Greece | |  |
|  |  | Greenland | |  |
|  |  | Guyanas South America†: | |  |
|  |  |  | French Guiana* |  |
|  |  |  | Guyana* |  |
|  |  |  | Suriname* |  |
|  |  | Guatemala | |  |
|  |  | Honduras | |  |
|  |  | Croatia | |  |
|  |  | Haiti | |  |
|  |  | Hungary | |  |
|  |  | Indonesia | |  |
|  |  | India | |  |
|  |  | Ireland | |  |
|  |  | Iran | |  |
|  |  | Iraq | |  |
|  |  | Iceland | |  |
|  |  | Israel | |  |
|  |  | Italy Plus: | |  |
|  |  |  | Italy* |  |
|  |  |  | Malta* |  |
|  |  |  | San Marino* |  |
|  |  |  | Vatican City* |  |
|  |  | Jamaica | |  |
|  |  | Jordan | |  |
|  |  | Japan | |  |
|  |  | Kazakhstan | |  |
|  |  | Kenya | |  |
|  |  | Kyrgyzstan | |  |
|  |  | Cambodia | |  |
|  |  | South Korea | |  |
|  |  | Laos | |  |
|  |  | Lebanon | |  |
|  |  | Liberia | |  |
|  |  | Libya | |  |
|  |  | Sri Lanka | |  |
|  |  | Lesotho | |  |
|  |  | Moldova | |  |
|  |  | Madagascar | |  |
|  |  | Mexico | |  |
|  |  | Mali | |  |
|  |  | Myanmar | |  |
|  |  | Mongolia | |  |
|  |  | Morocco Plus: | |  |
|  |  |  | Morocco* | 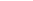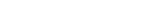 |
|  |  |  | Western Sahara* |  |
|  | 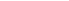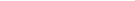 | Mozambique | |  |
|  |  | Mauritania | |  |
|  |  | Malawi | |  |
|  |  | Malaysia | |  |
|  |  | Namibia | |  |
|  |  | Niger | |  |
|  | 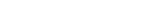 | Nigeria | |  |
|  |  | Nicaragua | |  |
|  |  | Netherlands | |  |
|  |  | Norway | |  |
|  |  | Nepal | |  |
|  |  | New Zealand | |  |
|  |  | Other Atlantic Ocean†: | |  |
|  |  |  | Bermuda* |  |
|  |  |  | Bouvet Island* |  |
|  |  |  | Cape Verde* |  |
|  |  |  | Falkland Islands* |  |
|  |  |  | Faroe Islands* |  |
|  |  |  | South Georgia and South Sandwich Islands* | |
|  |  |  | Saint Helena, Ascension, and Tristan de Cunha* | |
|  |  |  | Svalbard and Jan Mayen* | |
|  |  |  | Saint Pierre and Miquelon* | |
|  |  |  | Sao Tome and Principe* | |
|  |  | Other Balkans: | | 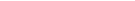 |
|  | 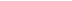 |  | Bosnia-Herzegovina* |  |
|  |  |  | 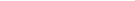Macedonia (FYR) * |  |
|  |  |  | Montenegro* |  |
|  |  |  | Serbia* |  |
|  |  | Other Indian Ocean†: | |  |
|  |  |  | Southern Territories* |  |
|  |  |  | Keeling Islands* |  |
|  | 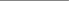 |  | Comoros* |  |
|  |  |  | Christmas Island* |  |
|  | 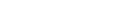 |  | Heard and McDonald Islands* | |
|  |  |  | British Indian Ocean Territory* | |
|  |  |  | Maldives* |  |
|  |  |  | Mauritius* |  |
|  |  |  | Mayotte* |  |
|  |  |  | Reunion* |  |
|  |  |  | Seychelles* |  |
|  |  | Other Pacific Ocean†: | |  |
|  |  |  | American Samoa* |  |
|  |  |  | Cook Islands* |  |
|  |  |  | Micronesia* |  |
|  |  |  | Guam* |  |
|  |  |  | Kiribati* |  |
|  |  |  | Marshall Islands* |  |
|  |  |  | Northern Mariana Islands* | |
|  |  |  | New Caledonia* |  |
|  |  |  | Norfolk Island* |  |
|  |  |  | Niue* |  |
|  |  |  | Nauru* |  |
|  |  |  | Pitcairn* |  |
|  |  |  | Palau* |  |
|  |  |  | French Polynesia* |  |
|  |  |  | Tokelau* |  |
|  |  |  | Tonga* |  |
|  |  |  | Tuvalu* |  |
|  |  |  | Minor Outlying Islands* | |
|  |  |  | Wallis and Futuna* |  |
|  |  |  | Samoa* |  |
|  |  | Other Southeast Asia: | |  |
|  |  |  | Brunei* |  |
|  |  |  | Singapore* |  |
|  |  | Pakistan | |  |
|  |  | Panama | |  |
|  |  | Peru | |  |
|  |  | Philippines | |  |
|  |  | Papua New Guinea | |  |
|  |  | Poland | |  |
|  |  | North Korea | |  |
|  |  | Portugal | |  |
|  |  | Paraguay | |  |
|  |  | Occupied Palestinian Territory | | |
|  |  | Rest of Arab Peninsula: | |  |
|  |  |  | United Arab Emirates* |  |
|  |  |  | Bahrain* |  |
|  |  |  | Kuwait* |  |
|  |  |  | Oman* |  |
|  |  |  | Qatar* |  |
|  |  | Romania | |  |
|  |  | Russia | |  |
|  |  | Rwanda | |  |
|  |  | Saudi Arabia | |  |
|  |  | Sudan Plus: | |  |
|  |  |  | Sudan* |  |
|  |  |  | South Sudan* |  |
|  |  | Senegal | |  |
|  |  | Solomon Islands | |  |
|  |  | Sierra Leone | |  |
|  |  | El Salvador | |  |
|  |  | Somalia | |  |
|  |  | Spain Plus: | |  |
|  |  |  | Andorra* |  |
|  |  |  | Spain* | 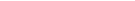 |
|  |  |  | Gibraltar* |  |
|  |  | Slovakia | |  |
|  |  | Slovenia | |  |
|  |  | Sweden | |  |
|  |  | Swaziland | |  |
|  |  | Syria | |  |
|  |  | Chad | |  |
|  | 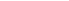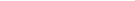 | Togo | |  |
|  |  | Thailand | |  |
|  |  | Tajikistan | |  |
|  |  | Turkmenistan | |  |
|  |  | Timor-L'este | |  |
|  |  | Tunisia | |  |
|  | 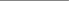 | Turkey | |  |
|  |  | Tanzania | |  |
|  | 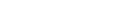 | Uganda | |  |
|  |  | Great Britain Plus: | |  |
|  |  |  | Great Britain* |  |
|  |  |  | Guernsey* |  |
|  |  |  | Isle of Man* |  |
|  |  |  | Jersey* |  |
|  |  | Ukraine | |  |
|  |  | Uruguay | |  |
|  |  | United States | |  |
|  |  | Uzbekistan | |  |
|  |  | Venezuela | |  |
|  |  | Vietnam | |  |
|  |  | Vanuatu | |  |
|  |  | Yemen | |  |
|  |  | South Africa | |  |
|  |  | Zambia | |  |
|  |  | Zimbabwe | |  |
